# Supplementary material for: Phospho‐regulation, nucleotide binding and ion access control in potassium‐chloride cotransporters
Source: EMBO J. 2021 May 25;40(14):e107294. doi: 10.15252/embj.2020107294 (PMC8280820; doi:10.15252/embj.2020107294)
Supplement: Supplementary file 1 — Appendix [file EMBJ-40-e107294-s004.pdf]

Appendix

Phospho-regulation, nucleotide binding and ion access control in K-Cl cotransporters

Gamma Chi[1,9*], Rebecca Ebenhoch[1,6,9*], Henry Man[1,8,9*], Haiping Tang[2,*], Laurence E. Tremblay[4], Gabriella Reggiano[3], Xingyu Qiu[2], Tina Bohstedt[1,9], Idlir Liko[7], Fernando G. Almeida[7], Alexandre P. Garneau[4,5], Dong Wang[1,9], Gavin McKinley[1,9], Christophe Moreau[1,10], Kiran D. Bountra[7], Patrizia Abrusci[1,8,9], Shubhashish M. M. Mukhopadhyay[1,9], Alejandra Fernandez-Cid[1,9], Samira Slimani[4], Julie L. Lavoie[5], Nicola Burgess-Brown[1,9], Ben Tehan[7], Frank DiMaio[3], Ali Jazayeri[7], Paul Isenring[4] , Carol V. Robinson[2,7], and Katharina L. Dürr[1,7,9†]

[1] Nuffield Department of Medicine, Centre of Medicines Discovery, University of Oxford, Roosevelt Drive, Oxford, OX3 7DQ, UK

[2] Physical and Theoretical Chemistry Laboratory, University of Oxford, South Parks Road, Oxford, OX1 3QZ, UK

[3] Department of Biochemistry, University of Washington, 1705 NE Pacific St, Seattle, 98195, Washington, USA

[4] Department of Medicine, Nephrology Research Group, Faculty of Medicine, Laval University, Quebec City, Québec, Canada

[5] Cardiometabolic Axis, School of Kinesiology and Physical Activity Sciences, University of Montréal, Montréal, Quebec, Canada

[6] Present address: MedChem, Boehringer Ingelheim Pharma GmbH & Co. KG, Birkendorferstrasse 65, 88397 Biberach, Germany

[7] Omass Therapeutics, Ltd., The Schrödinger Building, Heatley Road, Oxford Science Park, Oxford, OX4 4GE, UK.

[8] Present address: Exscientia Ltd, The Schrödinger Building, Heatley Road, The Oxford Science Park, Oxford, OX4 4GE.

[9] Structural Genomics Consortium, Nuffield Department of Medicine, University of Oxford, Roosevelt Drive, Oxford, OX3 7DQ, UK

[10] Present address: Celonic AG, Eulerstrasse 55, 4051 Basel, Germany

[*]these authors contributed equally to this work, listed in alphabetical order

[†]corresponding author, lead contact: katharina.duerr@omass.com

Table of Content

Appendix Figure S1: Secondary Structure Representation..4

Appendix Figure S2: Sequence Alignment 6

Appendix Figure S3: EM Processing Workflow for KCC1..9

Appendix Figure S4: EM Processing Workflow for KCC3...10

Appendix Figure S5: Determination of the N-terminal sequence by Rosetta MD…….......12

Appendix Figure S6: Phosphorylation site identification by proteomic analysis (KCC1)...14

Appendix Figure S7: Phosphorylation site identification by proteomic analysis (KCC3)...15

Appendix Figure S8: Rb+ Uptake for KCC3b variants...20

Appendix Figure S9: HDX-MS data for KCC3b-PKO and KCC3b-PM 22

Appendix Figure S10: Electrostatic representation of the nucleotide binding pocket……23

Appendix Figure S11: LC-MS data for KCC1...25

Appendix Figure S12: Molecular Dynamics results for KCC1 with bound ATP.................27

Appendix Figure S13: Molecular Dynamics results for KCC3 with bound ATP…..............28

Appendix Figure S14: NanoDSF data...30

Appendix Supplementary Methods…………………………..…………………………………….……..….31

References…….36

Secondary Structure Representation

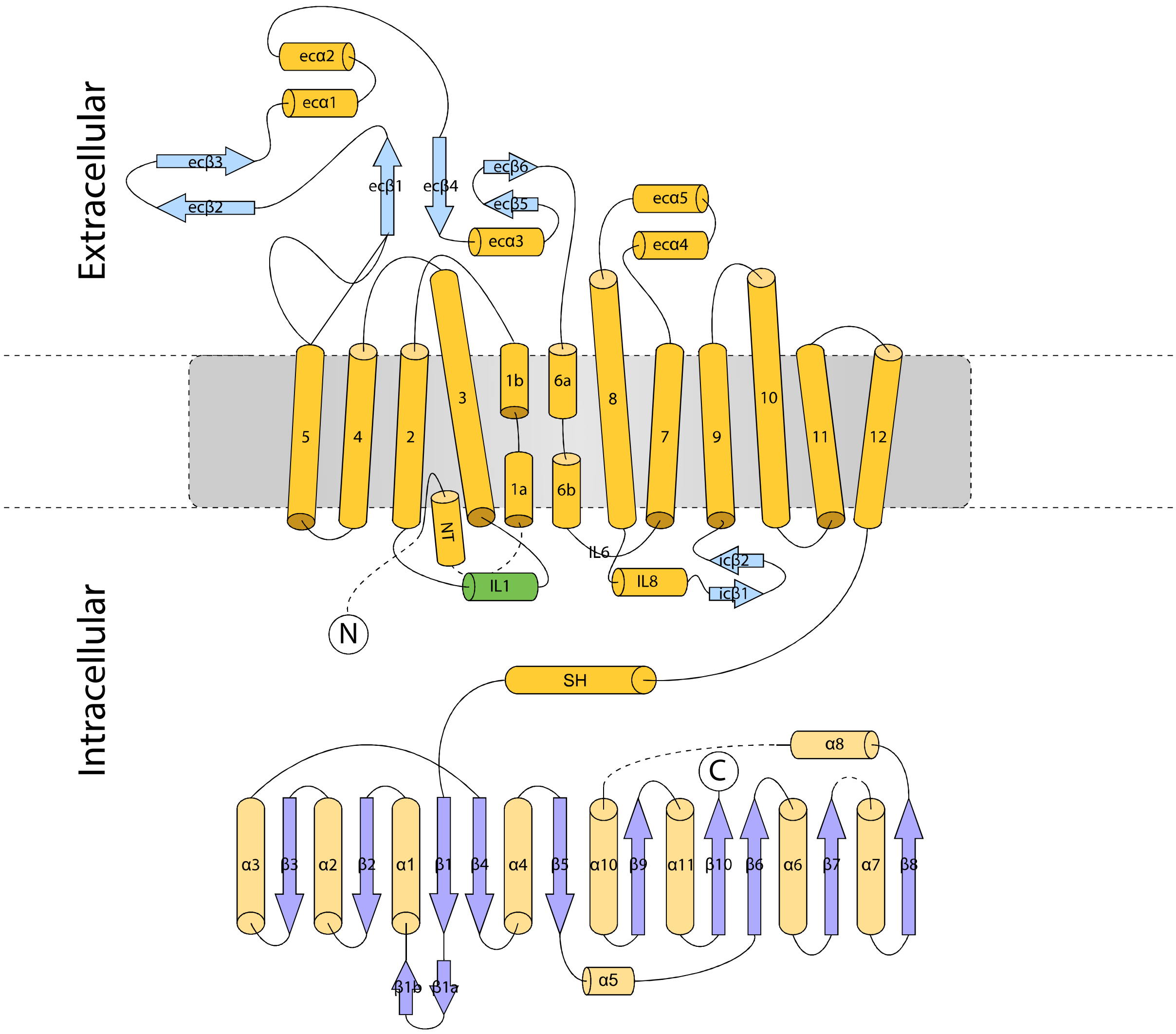

Appendix Figure S1 – Overall secondary structure depiction of both KCC1 and KCC3.

Sequence Alignment

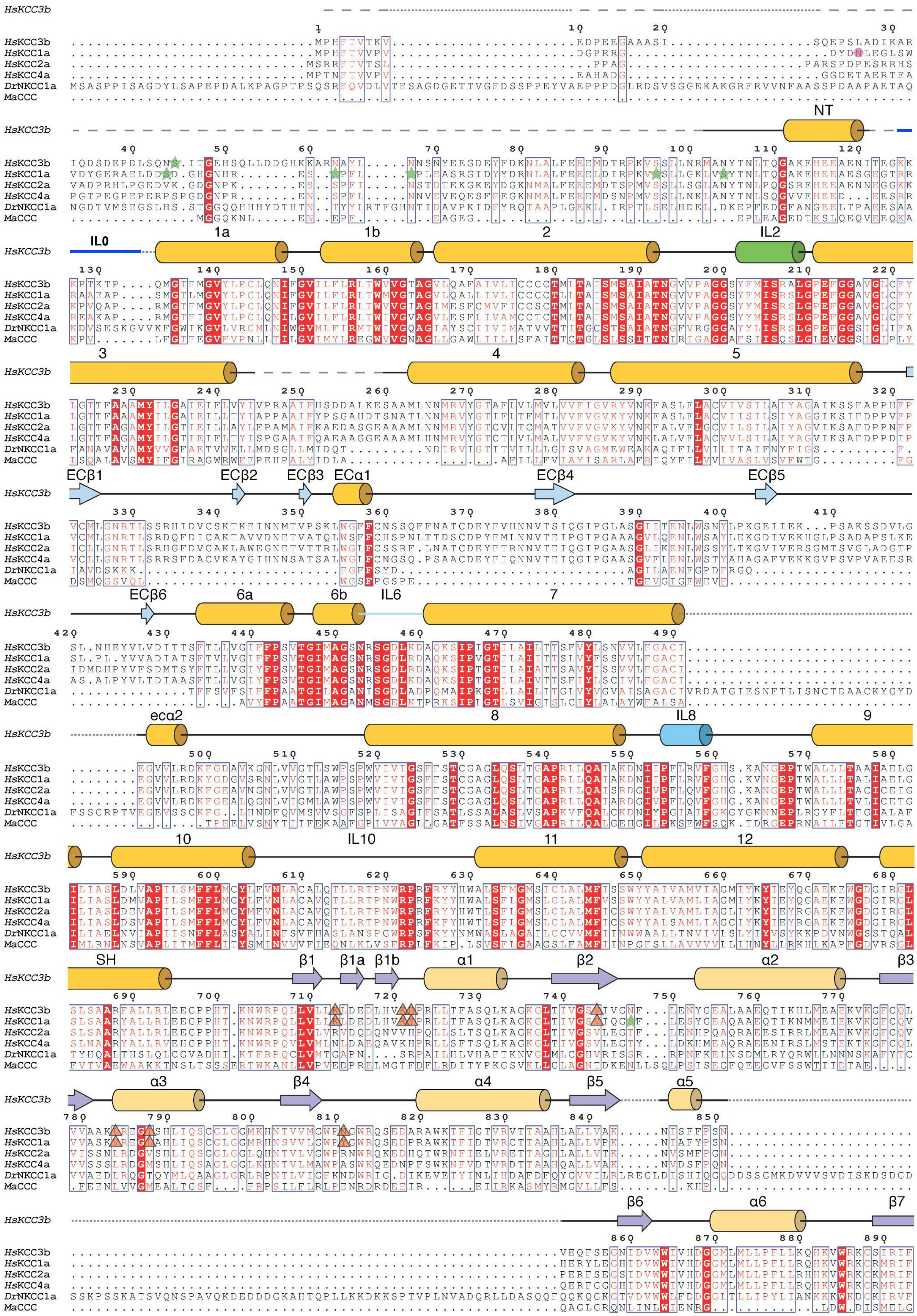
HsKCC3b
HsKCC1a
HsKCC2a
HsKCC4a
DrNKCC1a
MaCCC
NT
IL0
1a
1b
2
IL2
3
4
5
ECβ1
ECβ2
ECβ3
ECα1
ECβ4
ECβ5
ECβ6
6a
6b
IL6
7
ecα2
8
IL8
9
10
IL10
11
12
SH
β1
β1a
β1b
α1
β2
α2
β3
α3
β4
α4
β5
α5
β6
α6
β7

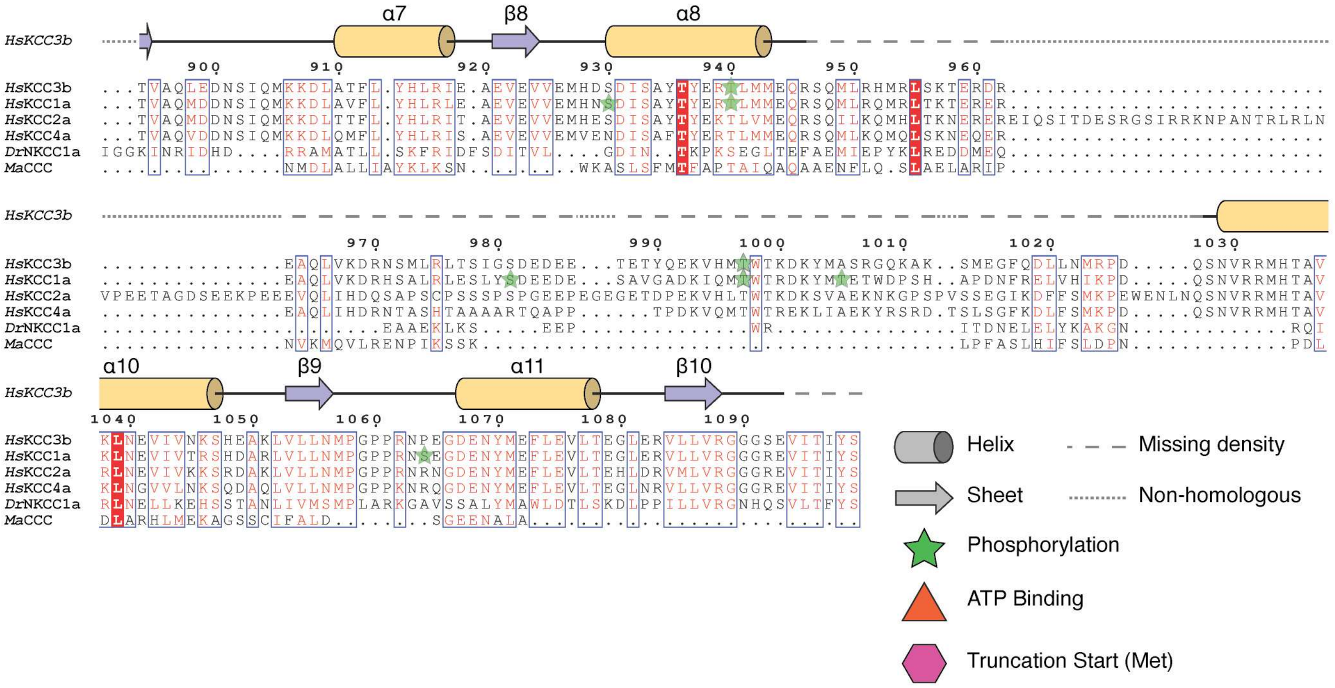

Appendix Figure S2 - Sequence alignment between *Hs*KCC1a,*Hs*KCC2a, *Hs*KCC3b, *Hs*KCC4a, *Dr*NKCC1 and *Ma*CCC using MUSCLE algorithm alignment with secondary structure prediction represented as yellow α-helices and purple β-strands, respectively.

EM Processing Workflows

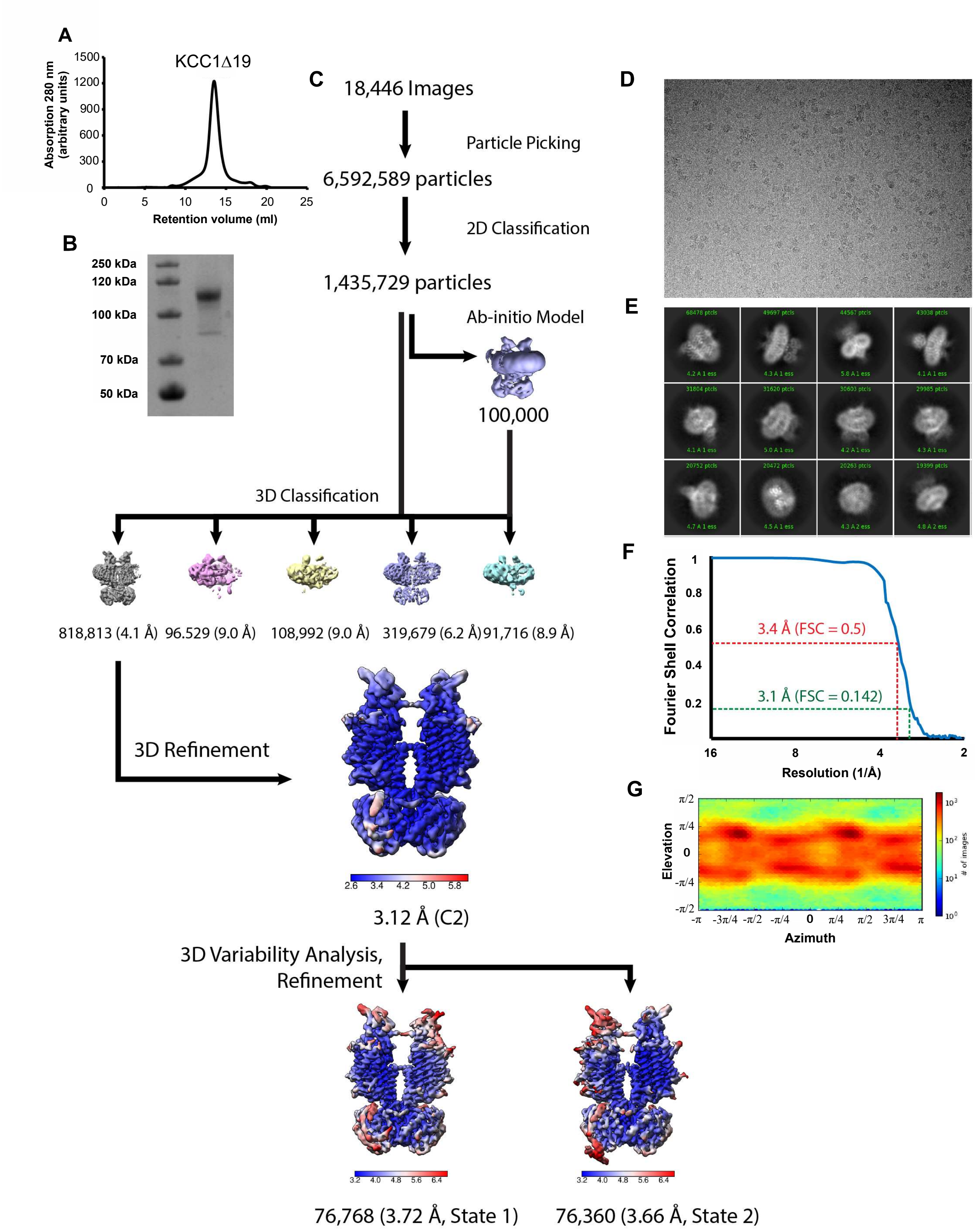

Appendix Figure S3 – (**A**) An example of SEC-purified KCC1 on a Superose 6 increase 10/300 GL column and (**B**) the SDS-PAGE analysis of the purified protein. (**C**) EM processing workflow for KCC1. (**D**) A typical micrograph and (**E**) the resulting 2D classification results for KCC1. (**F**) The Fourier shell correlation and (**G**) angular distribution obtained for the final reconstructions of KCC1.

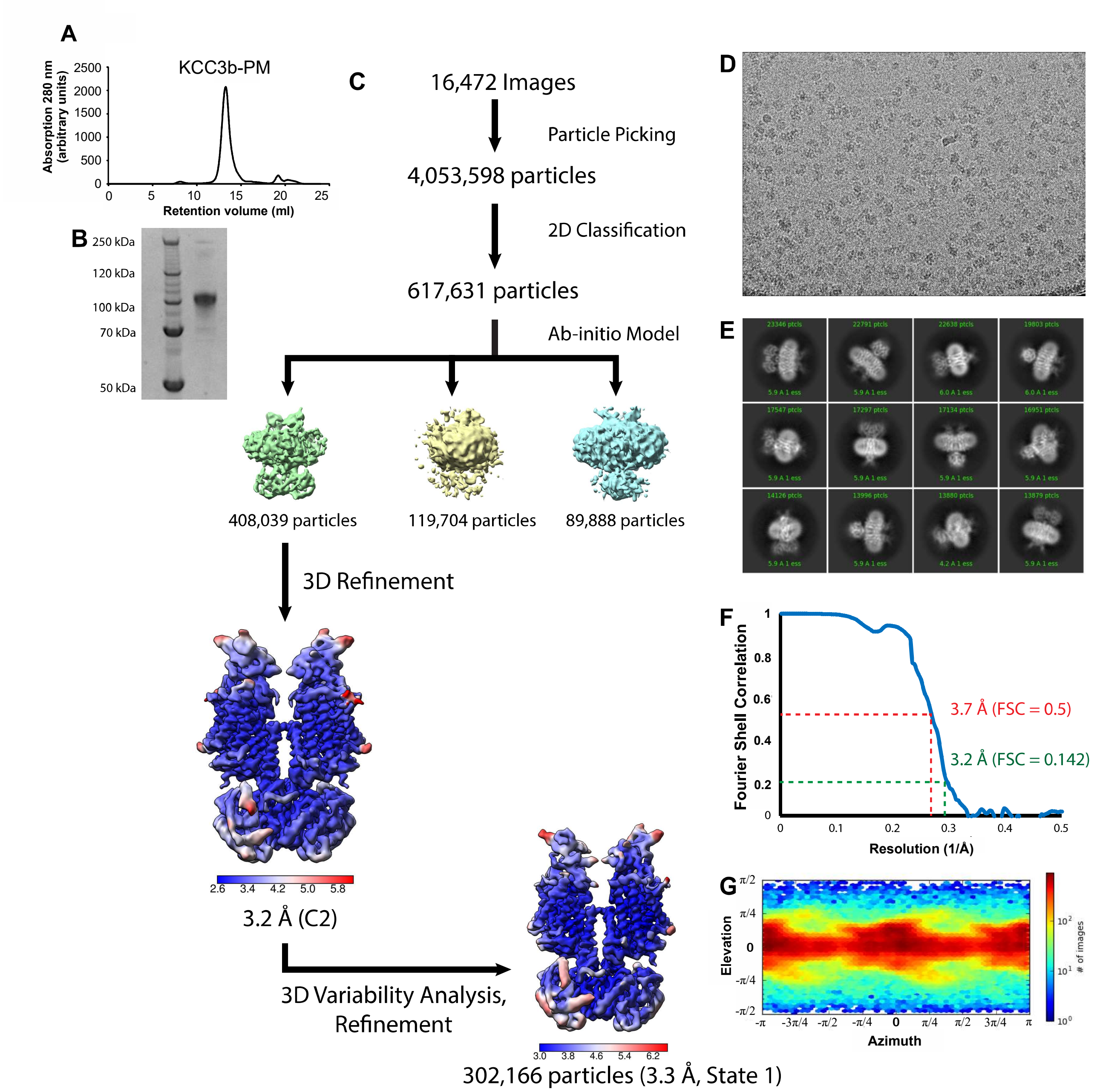

Appendix Figure S4 – (**A**) An example of size exclusion chromatography (SEC) purified KCC3b-PM on a Superose 6 increase 10/300 GL column and (**B**) the SDS-PAGE analysis of the purified protein. (**C**) EM processing workflow for KCC3b-PM. (**D**) A typical micrograph and (**E**) the resulting 2D classification results for KCC3. (**F**) The Fourier shell correlation and (**G**) angular distribution obtained for the final reconstructions of KCC3b-PM.

Rosetta MD Modelling: Determination of the N-terminal Sequence

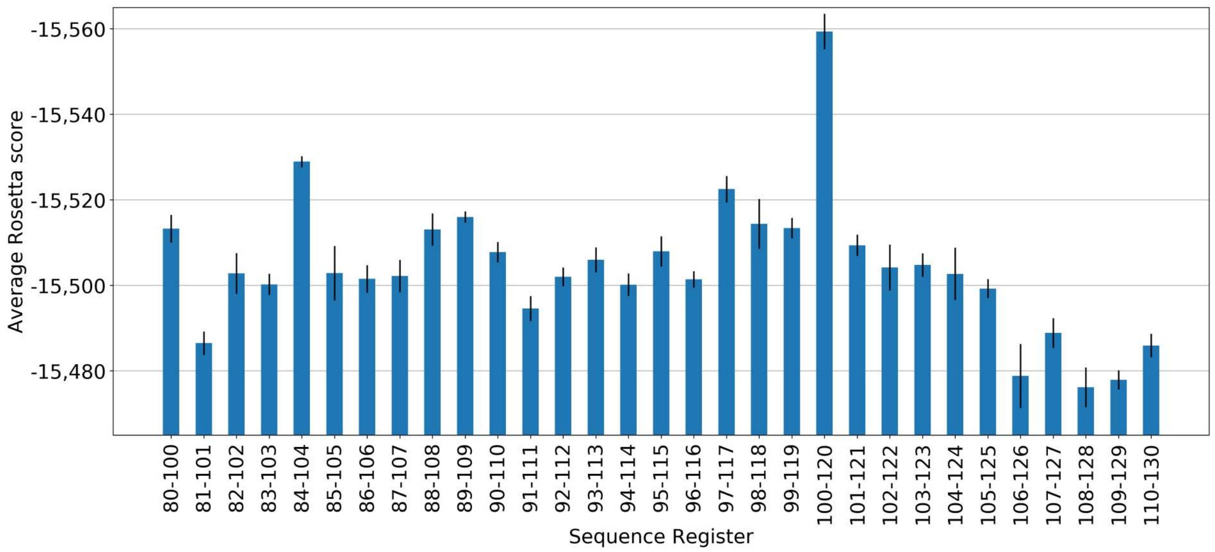

Appendix Figure S5 - Determination of the N-terminal sequence. N-terminal helix sequence identification using Rosetta score screen of 20-residue fragments. Error bars represent standard deviation calculated from ten independent trajectories of the FastRelax protocol.

Phosphorylation Site Identification by Proteomic Analysis (KCC1)

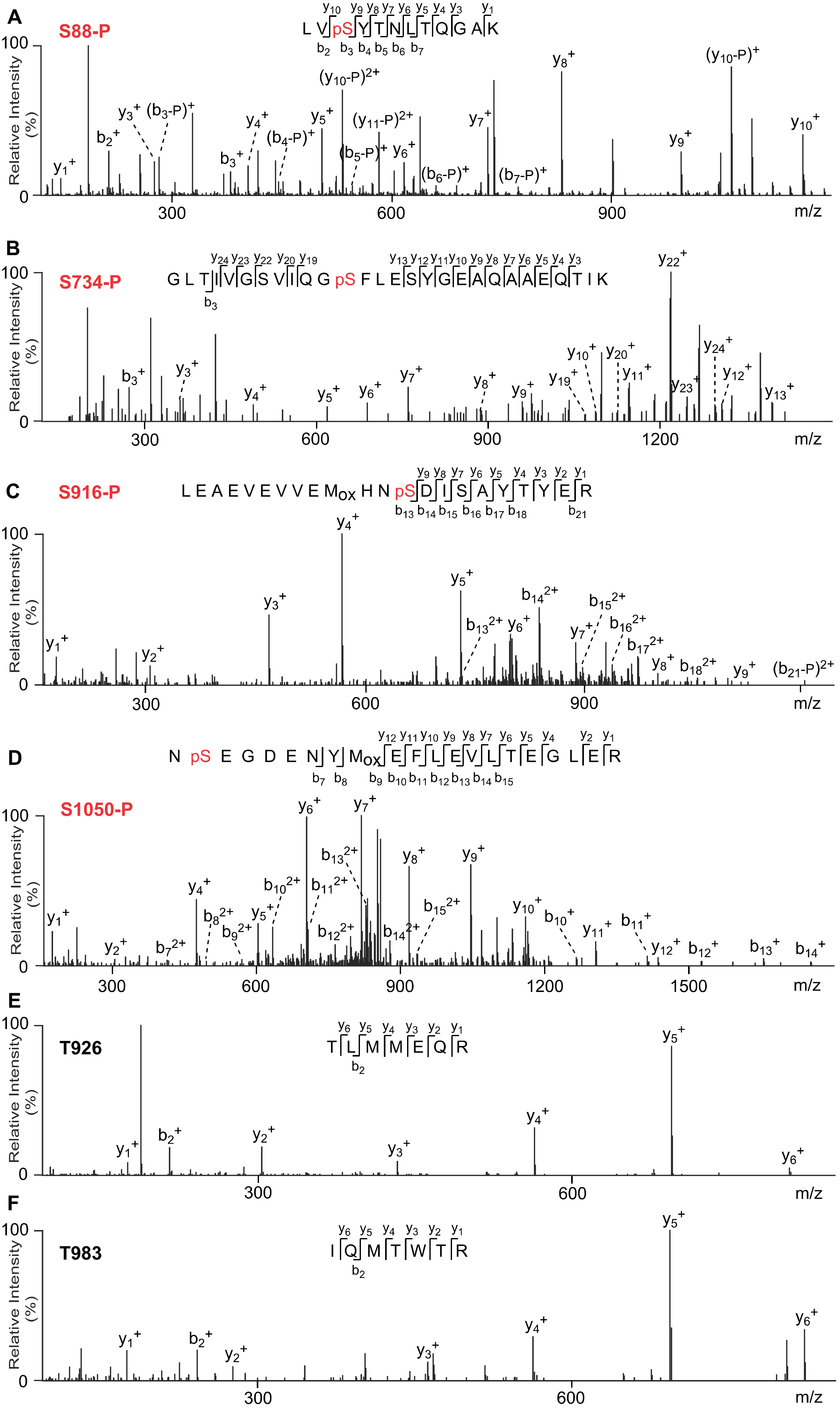
A
S88-P
L V pS Y T N L T Q G A K
B
S734-P
G L T I V G S V I Q G pS F L E S Y G E A Q A A E Q T I K
C
S916-P
L E A E V E V V E Mox H N pS D I S A Y T Y E R
D
N pS E G D E N Y Mox E F L E V L T E G L E R
S1050-P
E
T926
T L M M E Q R
F
T983
I Q M T W T R
Relative Intensity (%)
m/z

G

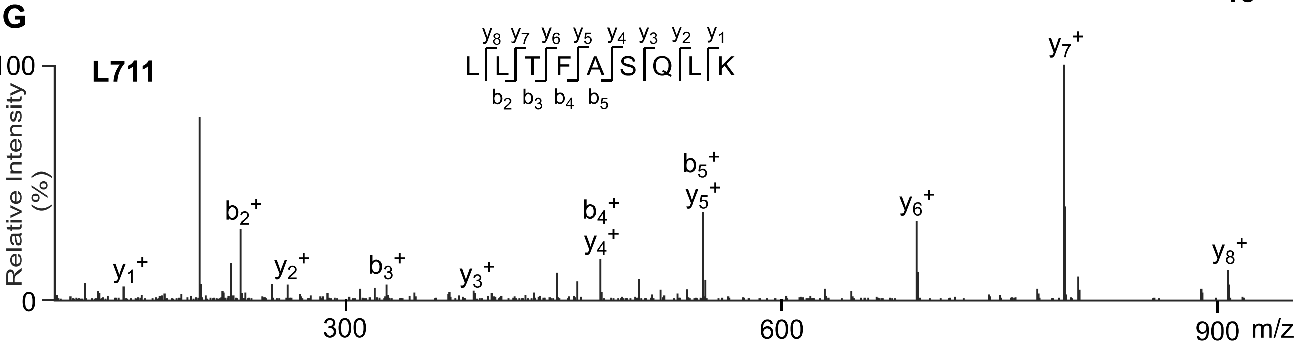

Appendix Figure S6 – Identification of phosphorylation sites of KCC1 by mass spectrometry. (**A**) The MS/MS spectrum of the mono-phosphorylated peptide, starting at Leu86, LVSYTNLTQGAK. The labeled peaks correspond to the masses of the b and y ions of the mono-phosphorylated peptide under the higher energy collision dissociation (HCD), indicating that phosphorylation is on the Ser-3 residue. (**B**) The MS/MS spectrum of the mono-phosphorylated peptide, starting at Gly-723, GLTIVGSVIQGSFLESYGEAQAAEQTIK. The labeled peaks correspond to the masses of the b and y ions of the mono-phosphorylated peptide, indicating that phosphorylation is on the Ser-12 residue. (**C**) The MS/MS spectrum of the mono-phosphorylated peptide, starting at Leu-904, LEAEVEVVEMHNSDISAYTYER in which the methionine was oxidized. The labeled peaks correspond to the masses of the b and y ions of the mono-phosphorylated peptide, indicating that phosphorylation is on the Ser-13 residue. (**D**) The MS/MS spectrum of the mono-phosphorylated peptide, starting at Asn1049, NSEGDENYMEFLEVLTEGLER in which the methionine was oxidized. The labeled peaks correspond to the masses of the b and y ions of the mono-phosphorylated peptide, indicating that phosphorylation is on the Ser-2 residue. (**E**) The MS/MS spectrum of the peptide, starting at Thr-926, TLMMEQR without phosphorylation. (**F**) The MS/MS spectrum of the peptide, starting at Ile-980, IQMTWTR without phosphorylation. (**G**) The MS/MS spectrum of the peptide, starting Leu-711, LLTFASQLK without phosphorylation.

Phosphorylation Site Identification by Proteomic Analysis (KCC3)

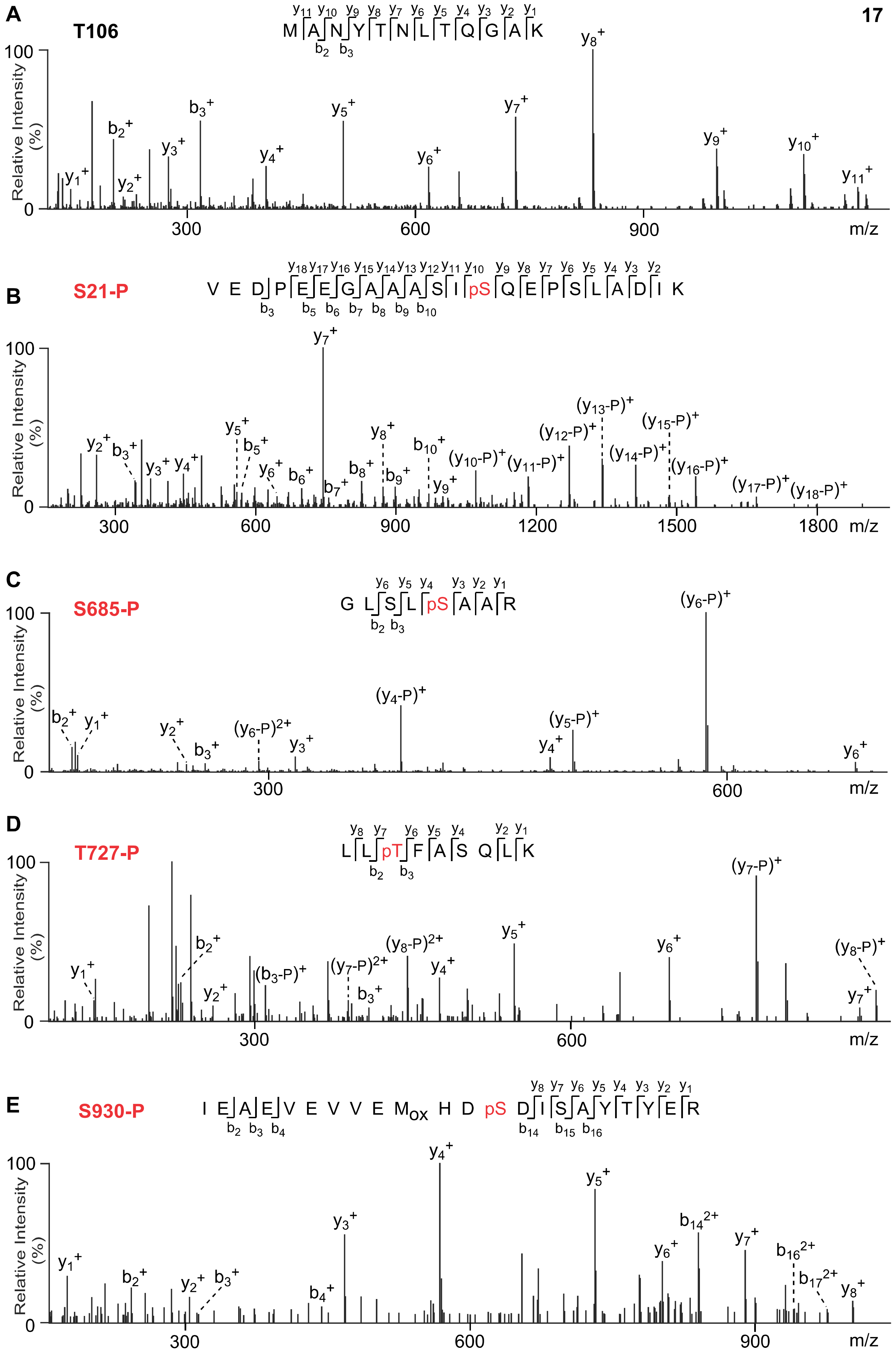
A
T106
M A N Y T N L T Q G A K
y11 y10 y9 y8 y7 y6 y5 y4 y3 y2 y1
b2 b3
Relative Intensity (%)
100
0
y1+ y2+ b2+ y3+ b3+ y4+ y5+ y6+ y7+ y8+ y9+ y10+ y11+
300
600
900
m/z
B
S21-P
V E D P E E G A A A S I pS Q E P S L A D I K
y18 y17 y16 y15 y14 y13 y12 y11 y10 y9 y8 y7 y6 y5 y4 y3 y2
b3 b5 b6 b7 b8 b9 b10
Relative Intensity (%)
100
0
y2+ b3+ y3+ y4+ y5+ b5+ y6+ b6+ y7+ b7+ b8+ y8+ b9+ y9+ b10+ (y10-P)+ (y11-P)+ (y12-P)+ (y13-P)+ (y14-P)+ (y15-P)+ (y16-P)+ (y17-P)+ (y18-P)+
300
600
900
1200
1500
1800
m/z
C
S685-P
G L S L pS A A R
y6 y5 y4 y3 y2 y1
b2 b3
Relative Intensity (%)
100
0
b2+ y1+ y2+ b3+ (y6-P)2+ y3+ (y4-P)+ y4+ (y5-P)+ (y6-P)+ y6+
300
600
m/z
D
T727-P
L L pT F A S Q L K
y8 y7 y6 y5 y4 y2 y1
b2 b3
Relative Intensity (%)
100
0
y1+ b2+ y2+ (b3-P)+ (y7-P)2+ b3+ (y8-P)2+ y4+ y5+ y6+ (y7-P)+ (y8-P)+ y7+
300
600
m/z
E
S930-P
I E A E V E V V E Mox H D pS D I S A Y T Y E R
y8 y7 y6 y5 y4 y3 y2 y1
b2 b3 b4 b14 b15 b16
Relative Intensity (%)
100
0
y1+ b2+ y2+ b3+ b4+ y3+ y4+ y5+ y6+ b14 2+ y7+ b16 2+ b17 2+ y8+
300
600
900
m/z

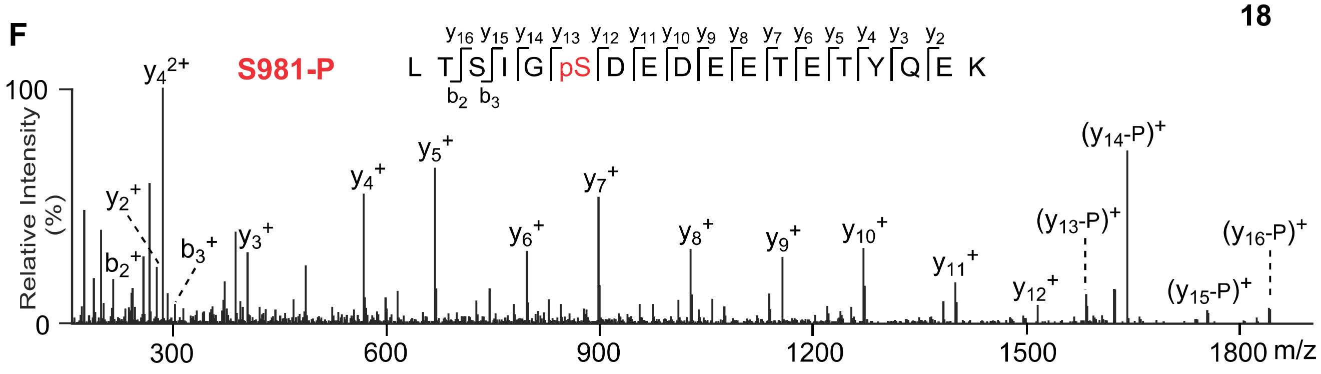

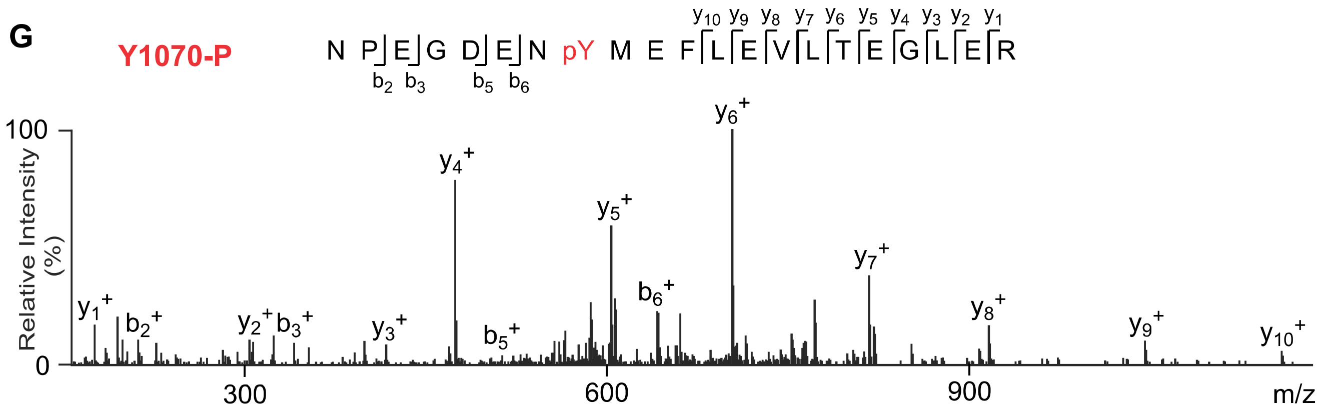

Appendix Figure S7 - Identification of phosphorylation sites of KCC3b_PM by mass spectrometry. (**A**) The MS/MS spectrum of peptide, starting at Met102, MANYTNLTQGAK.The labeled peaks correspond to the masses of the b and y ions of the peptide under the higher energy collision dissociation (HCD). (**B**) The MS/MS spectrum of the mono-phosphorylated peptide, starting at Val-9, VEDPEEGAAASISQEPSLADIK. The labelled peaks correspond to the masses of the b and y ions of the mono-phosphorylated peptide, indicating that phosphorylation is on the Ser-13 residue. (**C**) The MS/MS spectrum of the mono-phosphorylated peptide, starting at Gly-681, GLSLSAAR. The labeled peaks correspond to the masses of the b and y ions of the mono-phosphorylated peptide, indicating that phosphorylation is on the Ser-5 residue. (**D**) The MS/MS spectrum of the mono-phosphorylated peptide, starting at Leu-725, LLTFASQLK. The labeled peaks correspond to the masses of the b and y ions of the mono-phosphorylated peptide, indicating that phosphorylation is on the Thr-3 residue. (**E**) The MS/MS spectrum of the mono-phosphorylated peptide, starting at Ile-918, IEAEVEVVEMHDSDISAYTYER in which the methionine was oxidized. The labeled peaks correspond to the masses of the b and y ions of the mono-phosphorylated peptide, indicating that phosphorylation is on the Ser-13 residue. (**F**) The MS/MS spectrum of the mono-phosphorylated peptide, starting at Leu-976, LTSIGSDEDEETETYQEK. The labeled peaks correspond to the masses of the b and y ions of the mono-phosphorylated peptide, indicating that phosphorylation is on the Ser-6 residue. (**G**) The MS/MS spectrum of the mono-phosphorylated peptide, starting at Asn-1063, NPEGDENYMEFLEVLTEGLER. The labeled peaks correspond to the masses of the b and y ions of the mono-phosphorylated peptide, indicating that phosphorylation is on the Tyr-8 residue.

Rb^+ uptake for KCC3b variants

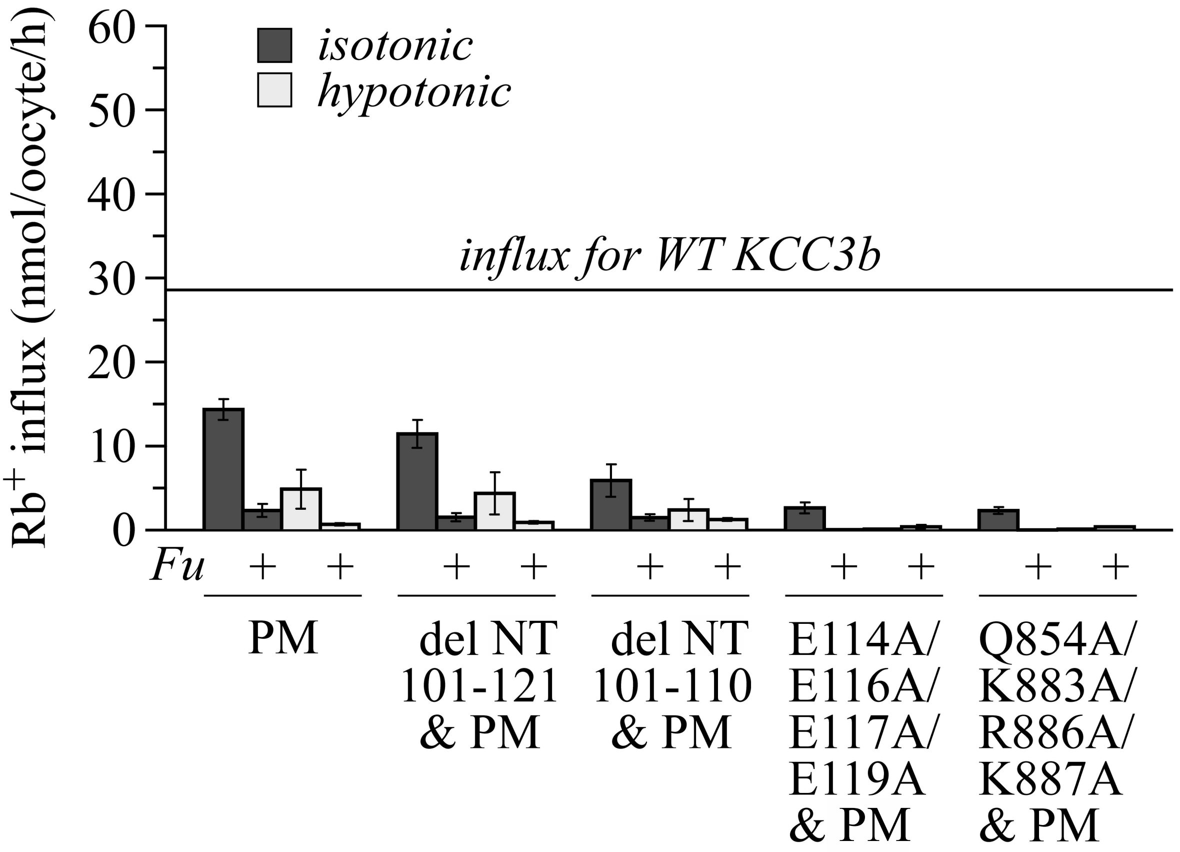

Appendix Figure S8 – Rb^+ uptake activity for KCC3b-PM and N-terminal and C-terminal mutants introduced into KCC3b-PM parent construct in comparison to KCC3b wild-type influx. Rb^+ uptake activities under isotonic (dark grey bars) and hypotonic conditions (grey bars) for the indicated del NT variants (residues 101-121 or residues 101-110 deleted) and charge-neutralizing mutations in the acidic helix of the N-terminus (E114A/E116A/E117A/E119A) or CTD (Q854A/K883A/R886A/K887A), all in the KCC3b-PM background. Average Rb^+ flux activity of wild-type KCC3b after hypotonic stimulation is indicated as horizontal line.

HDX-MS

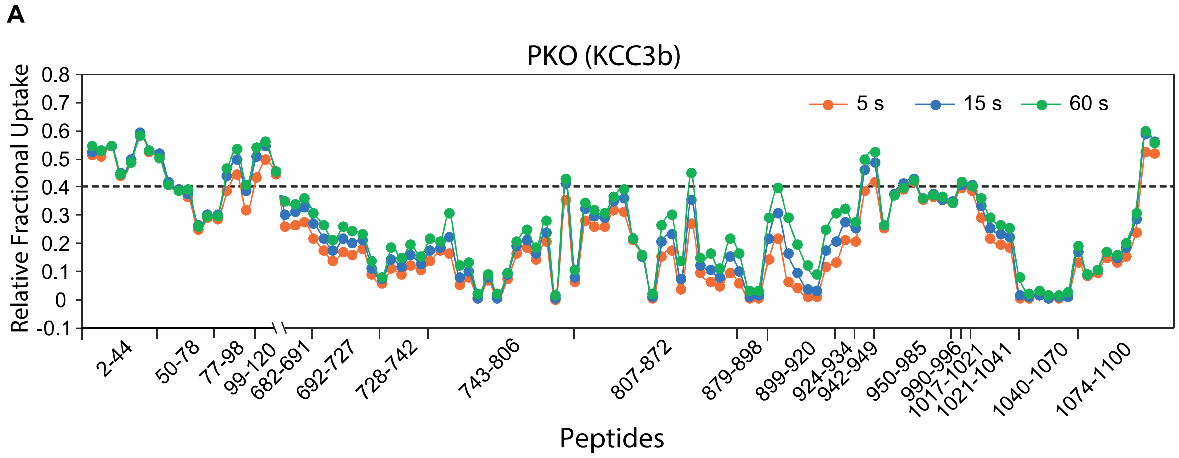

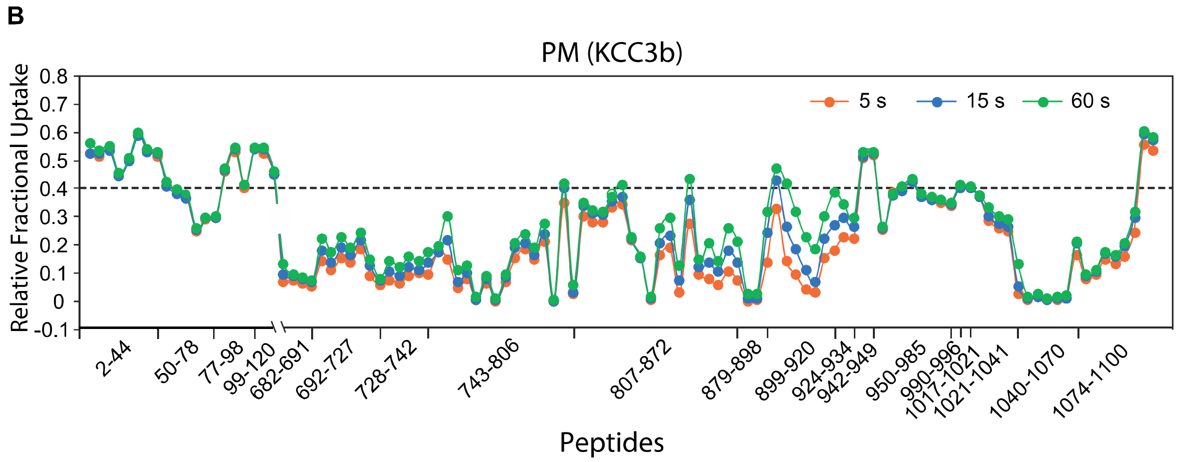

Appendix Figure S9- The relative fractional uptake of (**A**) KCC3b phospho-knockout (PKO) and (**B**) KCC3b phospho-mimetic (PM) constructs at three indicated time points are plotted against amino acid range covered by peptides. All data points are mean from triplicates.

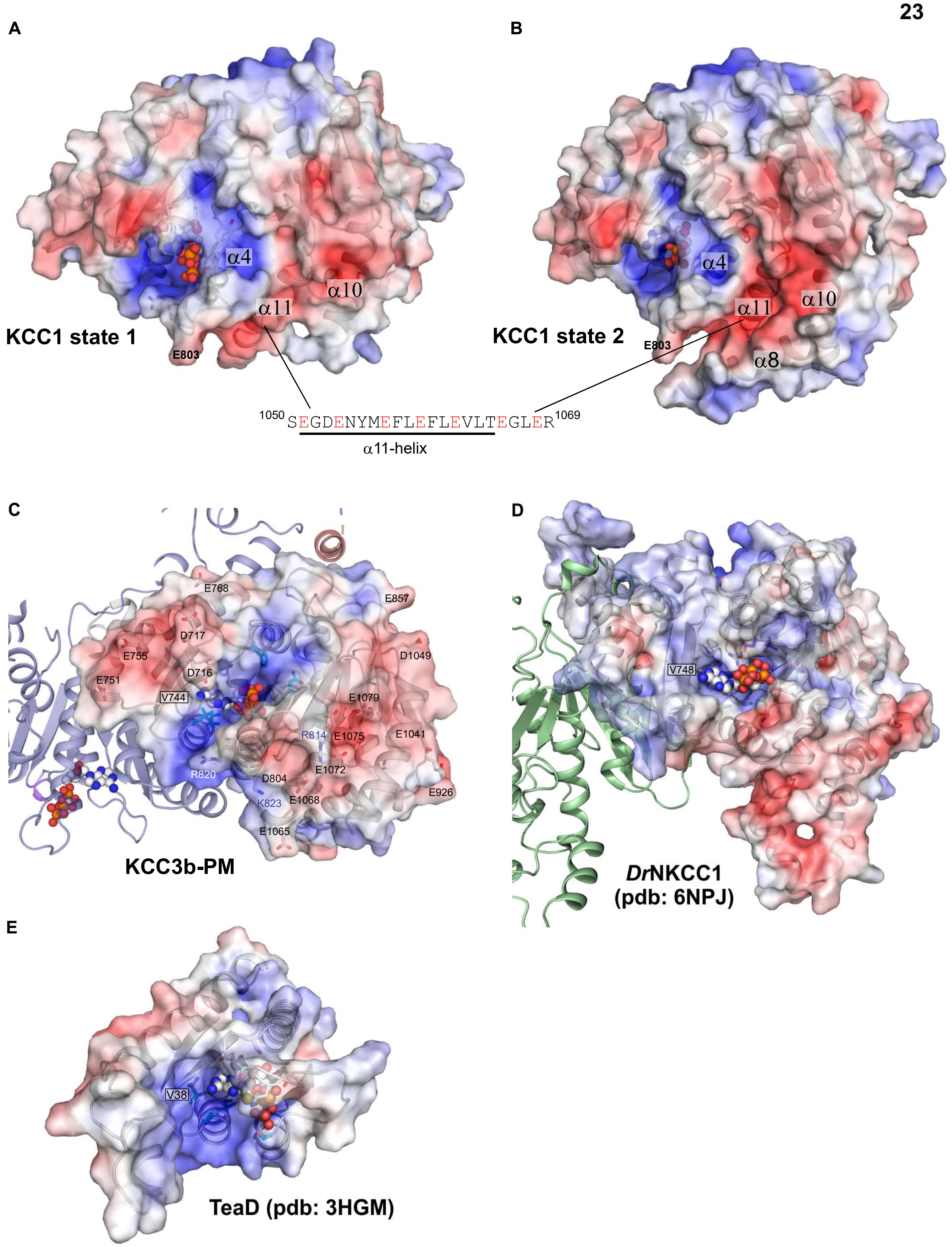

Appendix Figure S10 - Electrostatic surface presentation for CTD monomers with bound ATP (shown as spheres) for human KCC1, state 1 (**A**) and state B (**B**), which differ in the position of helix α8. In state 1, the α8 helix is located on the other side of α10 and α11. Highlighted is α11, which has a number of charged glutamate residues that are higly conserved within the potassium-transporting branch of the SLC12 family. **C-E**: Electrostatic surface presentation for CTD monomers with bound ATP (shown as spheres) for human KCC3b-PM (**C**), zebrafish NKCC1 (**D**) and for the regulatory subunit TeaD from *H.elongata* (**E**). The surface is coloured by electrostatic potential (red, $-5\,kT\,e^{-1}$; blue, $+5\,kT\,e^{-1}$).

LC-MS

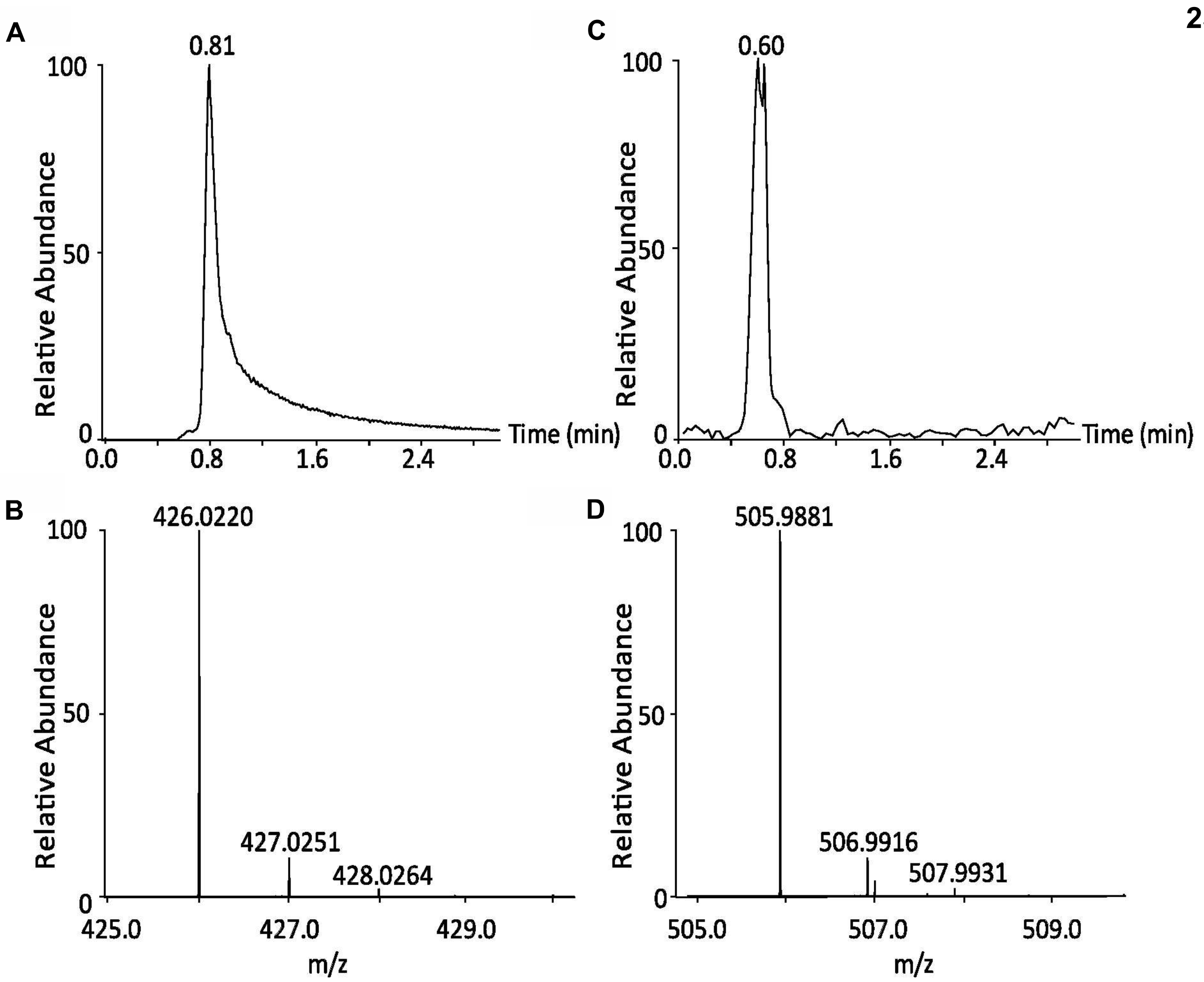

Appendix Figure S11 - LC-MS analysis of KCC1. (**A**) LC chromatogram after applying a mass filter using the theoretical mass of ADP and 5 ppm mass tolerance and (**B**) the resulted MS spectrum showing the presence of ADP in the sample. (**C**) LC chromatogram after applying a mass filter using the theoretical mass of ATP and 5 ppm mass tolerance and (**D**) the resulted MS spectrum showing the presence of ATP in the sample.

Molecular Dynamics Simulations

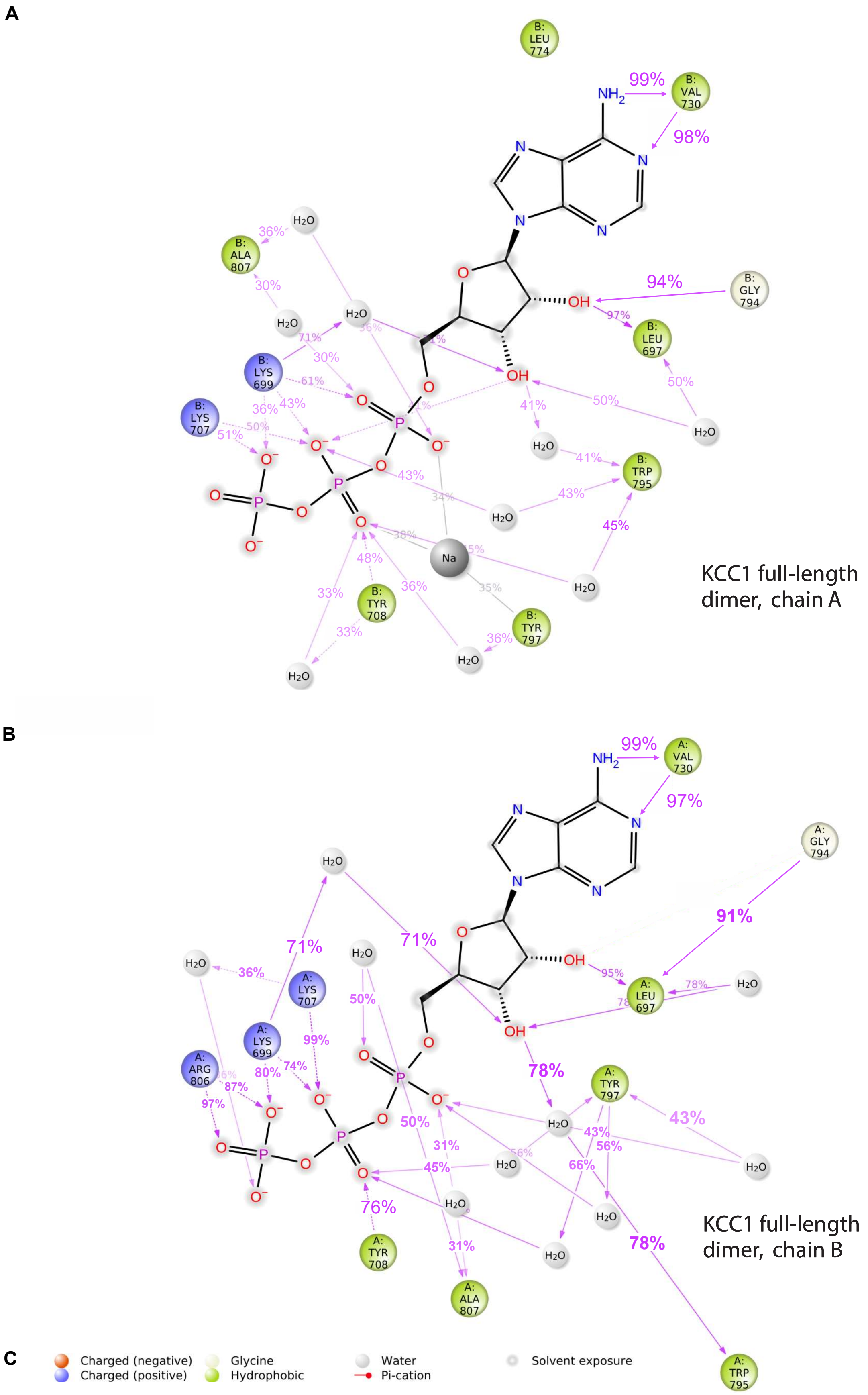

Appendix Figure S12 - MD simulations for ATP bound to KCC1Δ19 (full-length dimer). (**A**), Ligand protein interactions for ATP and chain A , inferred from a 300 ns MD run of the full-length KCC1Δ19 dimer. (**B**), Ligand protein interactions for ATP and chain B, inferred from a 300 ns MD run of the full-length KCC1Δ19 dimer.(**C**), Legend for protein ligand interactions shown in panels (**A**) and (**B**).

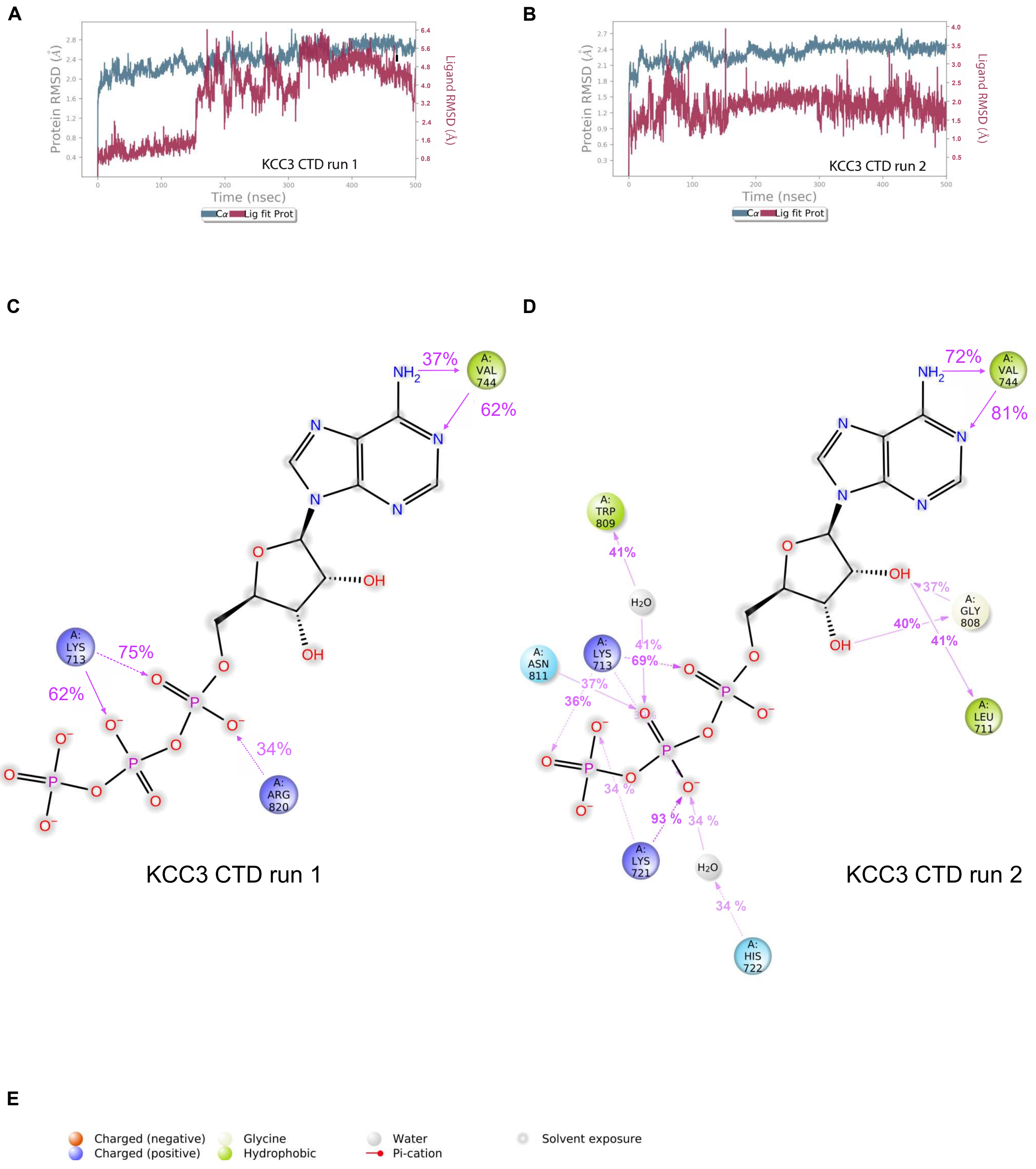

Appendix Figure S13 - MD simulations for ATP bound to KCC3 (monomeric CTD only). (**A**,**B**): Protein and ligand RMSD of ATP bound to the isolated KCC3b-PM CTD. RMSD for two independent runs over the course of a 500 ns simulation are shown in (A) and (B), respectively. (**C**,**D**) Ligand-protein contacts for ATP, inferred from two different 500 ns MD runs of the isolated KCC3b-PM CTD.(**E**) Legend for protein ligand interactions shown in panels (**C**) and (**D**).

NanoDSF Experiments

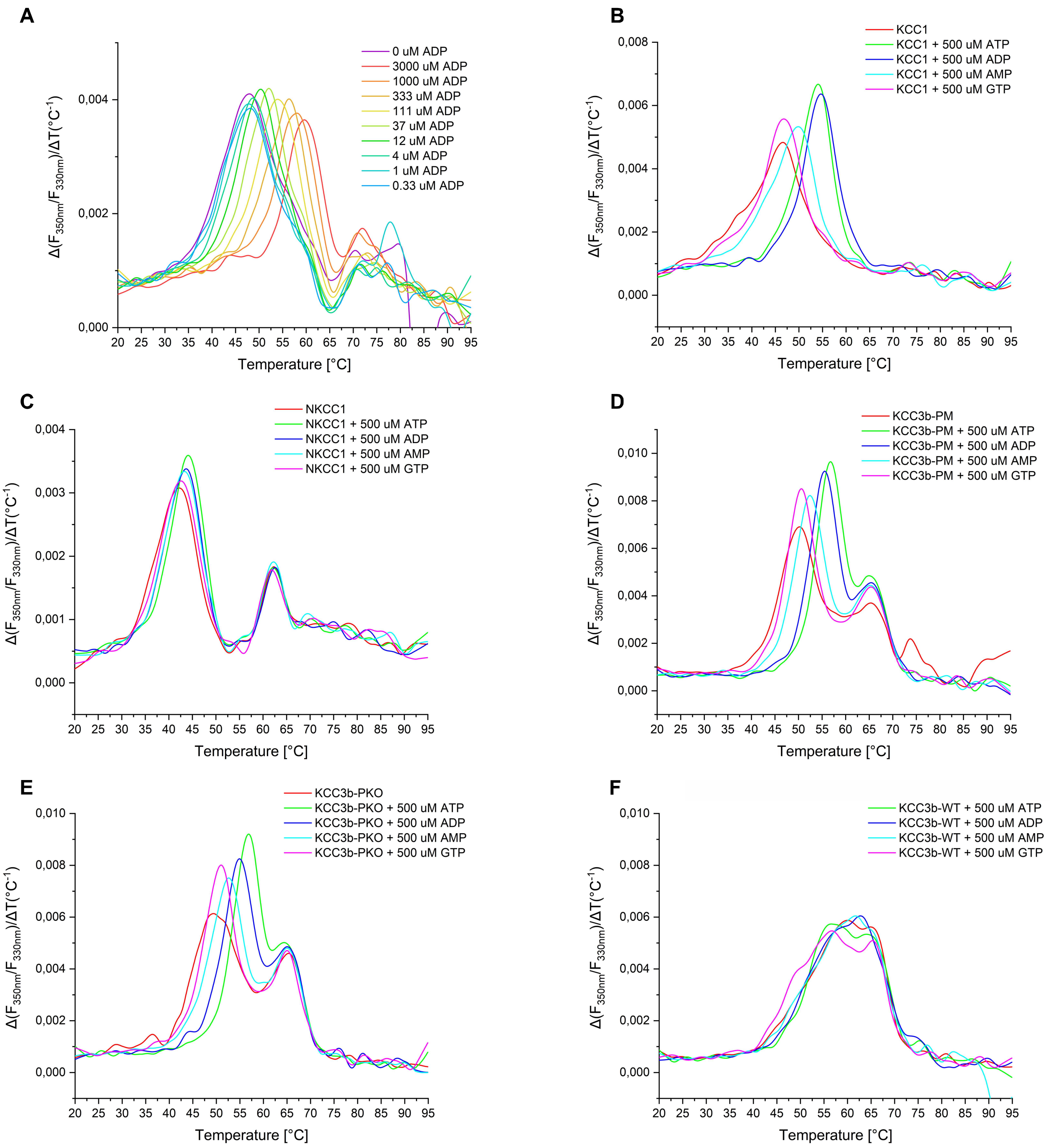

Appendix Figure S14 – First derivative of ratio between the intrinsic fluorescence signal at 330 and 350 nm indicate the melting behavior of protein samples. (**A**) KCC1 melting curves at different ADP (0-3 mM) concentrations show a concentrations dependent increase in melting temperature. Single concentration measurements (500 µM) of ATP (green), ADP (blue), AMP (teal) and GTP (magenta) in comparison to the apo protein (red) melting traces, shown for for KCC1 (**B**), NKCC1 (**C**), KCC3b_PM (**D**), KCC3b_PKO (**E**) and KCC3_WT (**F**).

Appendix Supplementary Methods

Molecular Biology, Virus Production and Protein Expression.

Full-length and Δ19-deleted human KCC1 (isoform A) and KCC3 isoform B were cloned from the mammalian gene collection (MGC: 1455 and 161519, IMAGE ID: 3349710 and 8991957, respectively) into LIC-adapted pHTBV C-terminally tagged twin-strep, 10-His vector with and without GFP. Full-length NKCC1, and phospho-mimetic (S45D, T940D, T997D) and phospho-knockout (S45A, T940A, T997A) constructs of KCC3b were synthesised (GenScript, Twist Bioscience) and subcloned into LIC-adapted pHTBV C-terminally tagged twin-strep, 10-His vector with GFP. The source clone for human NKCC1 is available from Addgene (plasmid ID #131908, ReSOLUTE collection).

Baculoviral DNA from the transformation of DH10Bac were used to transfect Sf9 cells to produce baculovirus particles for transduction. Virus was amplified by transducing mid-log Sf9 cells (2×10^6 cells mL^{-1}) grown in Sf-900™ II media supplemented with 2 % fetal bovine serum (Thermo Fisher Scientific). Cells were incubated on an orbital shaker for 65 h at 27 °C in 1 L shaker flasks. Baculovirus were harvested by centrifugation at 900*g* for 10 min with the virus contained in the supernatant.

1 L of Expi293F™ GnTI- cell cultures (2×10^6 cells mL^{-1}) in Freestyle 293™ Expression Medium (Thermo Fisher Scientific) were infected with high-titre P3 baculovirus (3 % v/v) in the presence of 5 mM sodium butyrate in a 2 L roller bottle (Biofil). Cells were grown in a humidity-controlled orbital shaker for 48 h at 37 °C with 8 % CO2 before being harvested by centrifugation at 900*g* for 10 min, washed with phosphate-buffered saline, and pelleted again prior to flash freezing in liquid nitrogen (LN2), then stored at −80 °C until needed.

Protein Purification of digitonin samples.

Whole cell pellets expressing varying constructs of NKCC1, KCC1, KCC2 and KCC3b were resuspended to a total volume of 50 mL per 15 g of cell pellet with buffer A (150 mM NaCl, 50 mM HEPES pH 7.5) supplemented with protease inhibitors 0.7 % w/v Lauryl Maltose Neopentyl Glycol, LMNG (Generon), and 0.07 % cholesteryl hemisuccinate, CHS (Generon). The cells were solubilised at 4 °C for 1 h with gentle

rotation. Cell debris was pelleted at 50,000*g* for 30 min. The clarified lysate was added to 0.5 mL bed volume of Strep-Tactin SuperFlow (IBA) per 50 mL of lysate, and allowed to bind at 4 °C for 1 h. The resin was collected on a gravity-flow column and washed with buffer B (buffer A with 0.003 % w/v LMNG and 0.0003 % w/v CHS), and then with buffer B supplemented with 1 mM ATP and 5 mM $MgCl_2$. Protein was eluted with 7 CV of buffer B supplemented with 50 mM D-desthiobiotin followed by tag-cleavage by TEV protease overnight and reverse purification. For LMNG/CHS condition, the samples were subjected to size exclusion chromatography pre-equilibrated with Buffer B. For digitonin condition, buffer A supplemented with 0.04 % digitonin (Apollo Scientific) was used for equilibration instead. Peak fractions were pooled and concentrated to 5 µM for LMNG/CHS sample and 50 µM for digitonin sample for subsequent experiments. Nanodisc sample was prepared similarly to the detergent samples with a few exceptions. After washing the protein-bound Strep-Tactin resin, buffer A supplemented with 0.5 % LMNG, 0.05% CHS and 0.125 % soy azolectin (Sigma) was added to a final LMNG concentration of 0.2 %, and purified MSP E3D1 protein to a final concentration of 0.5 mg/mL. The slurry was incubated on a rotating wheel for 15 min, then 100 mg of washed Biobead SM-2 per mL resin was added, followed by further incubation for a minimum of 4 hours. Subsequent purifications were then performed with buffer A.

Cryo-electron microscopy sample preparation, data collection and data processing.

All samples were frozen on Quantifoil Au R1.2/1.3 mesh 300 grids freshly glow discharged for 30 s, with plunge freezing performed on Vitrobot Mark IV (Thermo Fisher Scientific) chamber set to 80-100 % humidity and 4 °C. For LMNG/CHS and MSP E3D1 conditions, blotting time was set to 1.0 – 1.5 s, and for digitonin conditions it was set to 3.5 – 5.0 s after 30 s wait time.

The facilities and data collection strategies for each dataset is detailed in Table 1 and the data processing for digitonin datasets of KCC1 and KCC3b are detailed in Appendix Figures S3 and S4.

The potassium-free digitonin dataset of KCC3, collected at eBIC (Didcot, UK), was collected on a Titan Krios (Thermo Fisher Scientific) operating at 300 keV. 16,472

super-resolution dose-fractionated microcrographs (0.3255 Å $pixel^{-1}$) were collected on a K3 (Gatan) detector at 130,000 x nominal magnification by image shift collection two exposures per hole on SerialEM with a total dose of 45 e^{-} $Å^{-2}$ over 45 frames. Micrographs were binned to 0.651 Å $pixel^{-1}$ in relion 3.0.8 [1] during motion correction with 5 by 5 patches using MotionCor2 [2]. Motion-corrected images were then imported to Cryosparc 2.11.0 where defocus values were determined by Patch CTF function on Cryosparc. 4,053,596 particles were picked with blob picker function, and extracted particles were subjected to two cycles of 2D classification. 617,631 particles from good classes were used to generate three ab-initio models. 408,039 particles in the good model class were then used for non-uniform refinement function with C2 symmetry using the ab-initio model as reference. For 3D Variability Analysis (3DVA), the refined particles were symmetry-expanded to C1 space and a mask was generated from the unsharpened refined map. 3DVA was performed with four modes, and cluster function was used to isolate 302,166 particles for subclasses with longer α8 helix. These were then subjected to a new round of non-uniform refinement with C1 symmetry using the 3DVA reconstruction as reference. The potassium-saturated digitonin dataset of KCC3, collected at Midlands Regional Cryo-EM Facility (MRCEF; Leicester, UK), was collected on a Titan Krios operating at 300 keV; 4,731 super-resolution dose-fractionated micrographs (0.415 Å $pixel^{-1}$) were collected on a K3 detector at 105,000 x nominal magnification by aberration-free image shift (AFIS) collecting one exposure per hole on EPU with a total dose of 40 e^{-} $Å^{-2}$ over 45 frames. Micrographs were binned to 0.86 Å $pixel^{-1}$ in relion 3.0.8 during motion correction with 1 by 1 patches using MotionCor2 with defocus values determined by gCTF [3]. 1,471,447 particles were picked and extracted to 0.83 Å $pixel^{-1}$ with a box size of 300 pixel for 2D classifications and 3D classifications. 293,233 particles from good classes were selected then were subjected to 3D refinement with C1 symmetry. Iterations of per-particle CTF refinement and polishing were performed on the resulting map and particles until convergence in resolution was reached.

The potassium-free LMNG/CHS dataset of KCC3 was collected on a Titan Krios operating at 300 keV at ESRF (Grenoble, France). 3,155 movies in counting mode (1.067 Å $pixel^{-1}$) were collected on a K2 (Gatan) detector with two exposures per hole on EPU with total dose of 37.8 e^{-} $Å^{-2}$ over 40 frames. Micrographs were motion-corrected with full-frame motion correction on Cryosparc 2.11.0 and defocus values

determined with patch CTF function on Cryosparc. 1,582,396 particles were picked with blob picker function, and extracted particles were subjected to two cycles of 2D classification. 175,990 particles in good classes were used for 3D refinement in C1 symmetry with non-uniform refinement function on Cryosparc using the potassium-free digitonin map as reference.

The potassium-free MSP E3D1 dataset of KCC3 was collected on a Titan Krios operating at 300 keV at OPIC (Oxford, UK). 2,362 movies in counting mode (1.07 Å $pixel^{-1}$) were collected on a K2 detector with two exposures per hole on EPU with total dose of 52.5 e^{-} $Å^{-2}$ over 40 frames. Micrographs were imported to Relion 3.0.8 where they were motion-corrected with MotionCor2 and defocus values were determined by gCTF. 1,169,668 particles were picked with Laplacian of Gaussian picker function, and extracted particles were subjected to one round of 2D classification. 766,598 particles in good classes were exported to Cryosparc 2.11.0 and used for 3D refinement in C1 symmetry with non-uniform refinement function on Cryosparc using the potassium-free digitonin map as reference.

The potassium-free digitonin dataset of KCC1 was collected on a Titan Krios operating at 300 keV at MRCEF. 18,446 super-resolution dose-fractionated micrographs (0.425 Å $pixel^{-1}$) were collected on a K3 detector by aberration-free image shift (AFIS) collecting one exposure per hole on EPU with a total dose of 40 e^{-} $Å^{-2}$ over 45 frames. Micrographs were imported to Relion 3.0.8 where they were motion-corrected with MotionCor2 and defocus values were determined by gCTF. 6,592,589 particles were picked with Laplacian of Gaussian picker function, and subjected to one round of 2D classification. 4,283,713 particles were imported to Cryosparc 2.11.0 where they were subjected to two more rounds of 2D classification. 1,435,729 particles in good classes were then subjected to heterogeneous refinement with ab-initio model generated with 100,000 random particles was used as reference for five classes. 818,813 particles in the highest-resolution class was used for 3D refinement using non-uniform refinement function with C2 symmetry applied. For 3D Variability Analysis (3DVA), the refined particles were symmetry-expanded to C1 space and a mask was generated from the unsharpened refined map. 3DVA was performed with four modes, and cluster function was used to isolate particles for subclasses with alternative α8 helix positions (76,768 particles for subclass 1; 76,330 particles for subclass 2). These were then subjected

to a new round of non-uniform refinements with C1 symmetry using the 3DVA reconstructions as references.

Rosetta-assisted model generation

An initial model of the monomer was built by trimming the density map with Segger [4] in UCSF Chimera [5] to remove the detergent micelle. Homologous proteins (PDB: 6npl, 6npj, 6nph, 6irs, 6kkr) were manually docked into the density using Chimera's Fit in Map tool. The docked models and their alignments by hhpred [6] were given as templates to 3 rounds of iterative RosettaCM [7]. For each round, 250 models were generated. The Rosetta force field was augmented with a "fit to density" score term and the 5 lowest energy models were passed to the next round. The top model from iterative CM was split into two sub-structures, the TMD and CTD. Each sub-structure was then refined [8] as a separate domain in the corresponding multi-body refined map. Regions with poor agreement to the density were manually identified, trimmed and rebuilt using RosettaES [9]. Each domain was docked into the density of the remaining monomer and refined separately using Rosetta's FastRelax protocol [10]. Finally, individual domain models were recombined with RosettaCM to get both monomers and then refined as an asymmetric dimer in a composite map.

To find the sequence identity of the N-terminal helix, an idealized helix was manually fit into the multi-body refined map of the TMD in Chimera. Using Rosetta's *partial_thread* application, models of all possible sequence threadings with a length of 9 from residues 1 to 130 were threaded onto the backbone of the small helix and combined with the existing model for the TMD. Interacting residues of the TMD with the small helix were manually identified in Chimera. Each model was refined using Rosetta's relax protocol, where refinement was restricted only to the small helix and the interacting residues. 10 models were generated for each sequence threading and the thread with the lowest average score was used as a starting point to build the rest of the backbone with RosettaES. 11 additional residues were built with RosettaES. Models of all possible sequence threadings of length 20 from residues 1 to 130 were threaded onto the new backbone and combined with the TMD. Interacting residues of the TMD with the entire peptide were manually identified. Each model was refined with Rosetta's relax protocol, which was restricted to the threaded model and the

interacting residues of the TMD. Ten models were generated for each thread, and the lowest scoring model was selected.

Model building and refinement

A combination of the Rosetta generated model and a monomer model of *Dr*NKCC1's TMD (PDB: 6nph) was generated, and the sequence was substituted with KCC3's TMD sequence using CHAINSAW [11] were used as templates for manual building in Coot [12]. Models were refined using Phenix real space refine [13] and geometry of the models were verified in Phenix (MolProbity) [14]. The model for KCC3 was used as reference for KCC1 and sequence substitution was performed with CHAINSAW. Manual building and refinement was performed in Coot. Models were refined using Phenix real space refine, and geometry of the models were verified in Phenix (MolProbity).

References

1. Zivanov, J., et al., *New tools for automated high-resolution cryo-EM structure determination in RELION-3.* Elife, 2018. **7**.
2. Zheng, S.Q., et al., *MotionCor2: anisotropic correction of beam-induced motion for improved cryo-electron microscopy.* Nat Methods, 2017. **14**(4): p. 331-332.
3. Zhang, K., *Gctf: Real-time CTF determination and correction.* J Struct Biol, 2016. **193**(1): p. 1-12.
4. Pintilie, G.D., et al., *Quantitative analysis of cryo-EM density map segmentation by watershed and scale-space filtering, and fitting of structures by alignment to regions.* J Struct Biol, 2010. **170**(3): p. 427-38.
5. Goddard, T.D., C.C. Huang, and T.E. Ferrin, *Visualizing density maps with UCSF Chimera.* J Struct Biol, 2007. **157**(1): p. 281-7.
6. Soding, J., *Protein homology detection by HMM-HMM comparison.* Bioinformatics, 2005. **21**(7): p. 951-60.
7. Song, Y., et al., *High-resolution comparative modeling with RosettaCM.* Structure, 2013. **21**(10): p. 1735-42.
8. Wang, R.Y., et al., *Automated structure refinement of macromolecular assemblies from cryo-EM maps using Rosetta.* Elife, 2016. **5**.
9. Frenz, B., et al., *RosettaES: a sampling strategy enabling automated interpretation of difficult cryo-EM maps.* Nat Methods, 2017. **14**(8): p. 797-800.
10. Conway, P., et al., *Relaxation of backbone bond geometry improves protein energy landscape modeling.* Protein Sci, 2014. **23**(1): p. 47-55.
11. Stein, N., *CHAINSAW: a program for mutating pdb files used as templates in molecular replacement.* Journal of applied crystallography, 2008. **41.3**: p. 641-643.
12. Emsley, P. and K. Cowtan, *Coot: model-building tools for molecular graphics.* Acta Crystallogr D Biol Crystallogr, 2004. **60**(Pt 12 Pt 1): p. 2126-32.
13. Afonine, P.V., et al., *Real-space refinement in PHENIX for cryo-EM and crystallography.* Acta Crystallogr D Struct Biol, 2018. **74**(Pt 6): p. 531-544.
14. Klaholz, B.P., *Deriving and refining atomic models in crystallography and cryo-EM: the latest Phenix tools to facilitate structure analysis.* Acta Crystallogr D Struct Biol, 2019. **75**(Pt 10): p. 878-881.
