# Supplementary material for: Phospho‐regulation, nucleotide binding and ion access control in potassium‐chloride cotransporters
Source: EMBO J. 2021 May 25;40(14):e107294. doi: 10.15252/embj.2020107294 (PMC8280820; doi:10.15252/embj.2020107294)
Supplement: Supplementary file 2 — Expanded View Figures PDF [file EMBJ-40-e107294-s002.pdf]

Expanded View Figures

Figure EV1. TMD and CTD arrangement in KCC1Δ19 and KCC3b-PM, in comparison to *Dr*NKCC1 and CTD-free KCC1 (Related to Fig 1).

A Cartoon representation of the KCC3b-PM dimer with inset illustrating the TMD of one subunit. The transporter core domain is shown in orange, the scaffold domain in wheat.

B, C Slab view of the transporter domain of KCC3 (construct: PM) (B) and KCC1 (construct: Δ19) (C) in surface representation, highlighting the inward-open conformations with intracellular accessible vestibules.

D, E Extracellular view of the TMD dimer of KCC3b-PM (D) and *Dr*NKCC1 (E).

F, G Extracellular view onto central TM11/TM12 helices and CTD, highlighting counter-clockwise twisted CTD for KCC3b-PM (F) and clockwise twisted CTD for *Dr*NKCC1 (G).

H Extracellular view onto TMD dimer illustrating the alternative dimer interface observed in CTD-free KCC1 (6KKR).

I Front view on CTD-free KCC1 dimer detailing molecular interactions (inset) between IL8 (blue helix) and TM12 characteristic for the configuration in 6KKR.

© 2021 The Authors

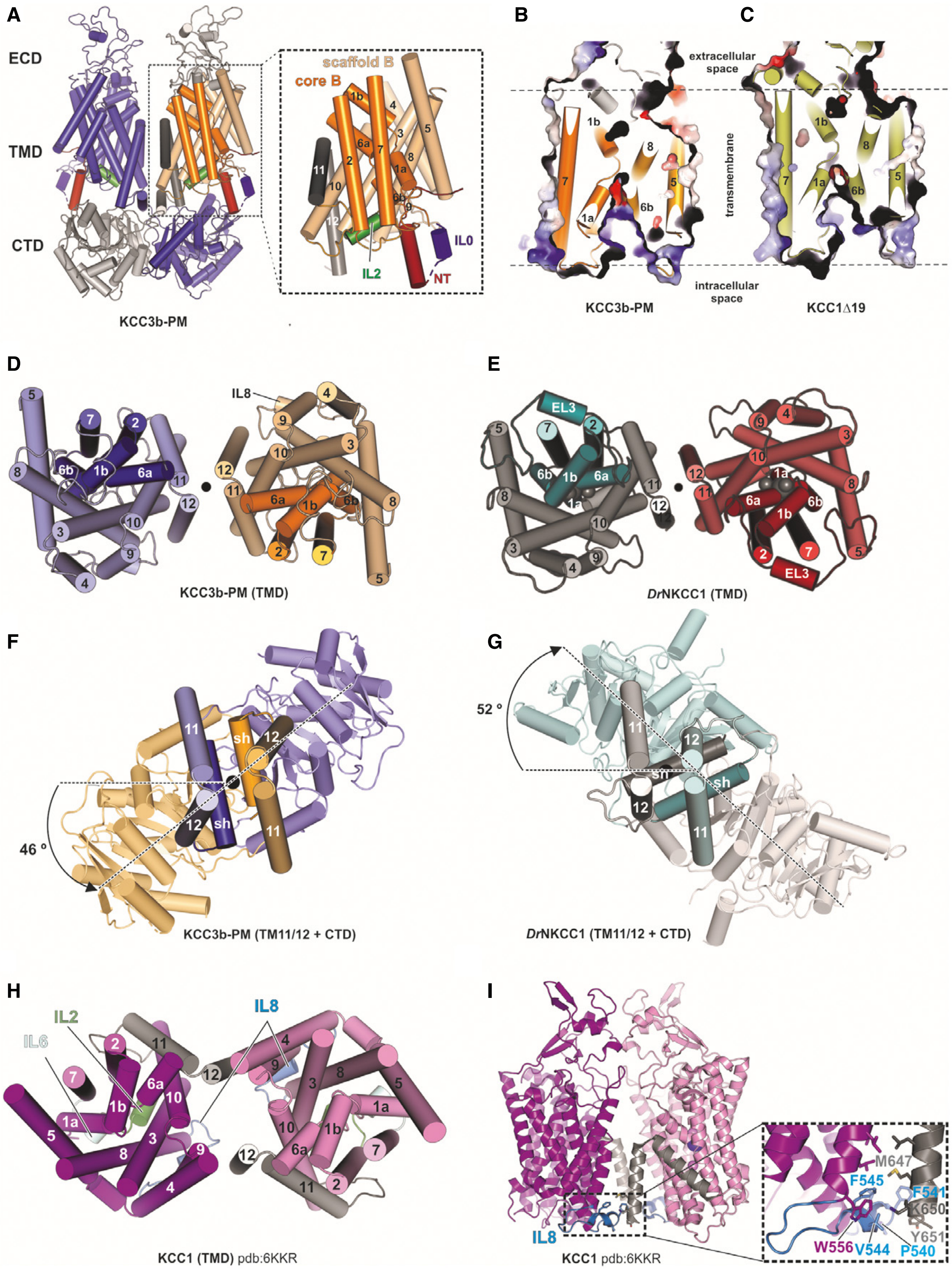

Figure EV1.

 © 2021 The Authors

Figure EV2. Detailed comparison of CTD-TMD interface of KCC3b and KCC1 structures (Related to Fig 2).

A–D Cryo-EM maps of KCC3b constructs determined in various conditions. Density for N-terminal helix (purple cartoon) is visible in the cryo-EM maps of KCC3b-PM in detergent environments, in both potassium-free (A) and potassium-saturated (D) conditions. On the other hand, there is no density for the N-terminal helix in the cryo-EM maps of KCC3b-WT in both detergent (B) and nanodisc (C) environments.

E, F Comparison of KCC3b-PM and KCC1Δ19 at the cation-binding site. Density for inorganic ion is not present in the KCC3b-PM map (E), but it is present in KCC1Δ19 (F), suggesting its inverse correlation with the presence of N-terminal segment.

G–J Differences in TMD/CTD interfaces between human KCC3b-PM (G, H) and *Dr*NKCC1 (I, J) as consequence of counter-clockwise and clockwise interdomain twist which brings either N-terminus (KCC3b) or C-terminus (*Dr*NKCC1) in close proximity to IL2. H: black frames highlight residues mutated to Ala for functional characterization in Appendix Fig S8.

© 2021 The Authors

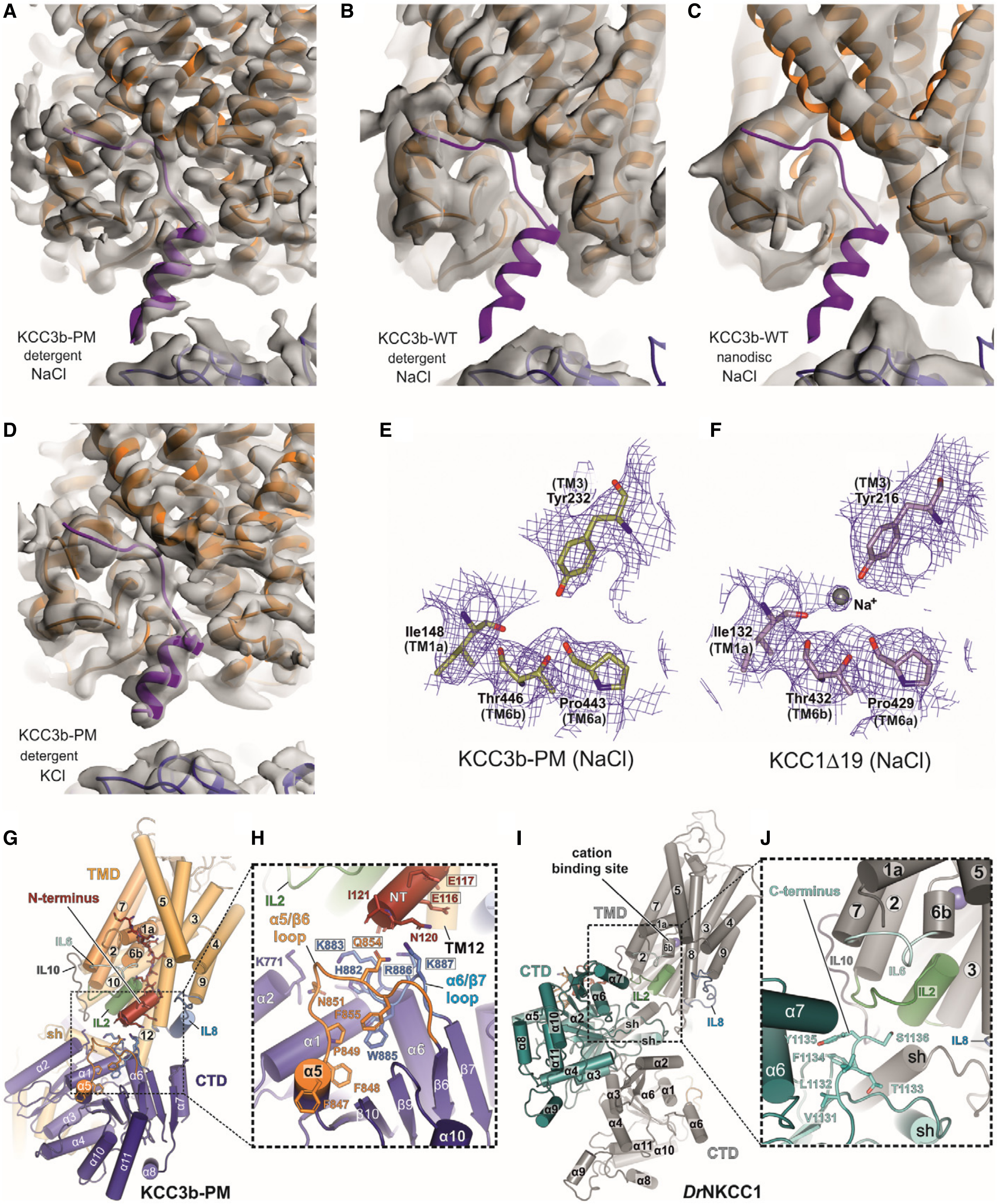

Figure EV2.

 © 2021 The Authors

Figure EV3. Rigid body groups and modes of movement in KCC1 and KCC3 (Related to Fig 3).

A Rigid body groups (oval circles) identified by 3D variability analyses of KCC1 and KCC3.

B–E The two man modes of movement are represented by red (mode 0) and blue (mode 1) arrows. The extent of each movement is indicated by a numerical value (in either ° or Å) in blue/yellow for KCC1Δ19 and purple/orange for KCC3b-PM. (B-C): redisplay of Fig 3B and C. (B): front view, (C): side view, (D): cytoplasmic view, (E): extracellular view.

F Alternative α8 conformation in the CTD of KCC1 from 3D variability analysis (Fig 3D), highlighting residues involved in interactions stabilizing this conformation (state 2).

G α8 arrangement in *Dr*NKCC1 structure, showing similarities to the helical positioning in KCC1, state 2 (panel F).

© 2021 The Authors

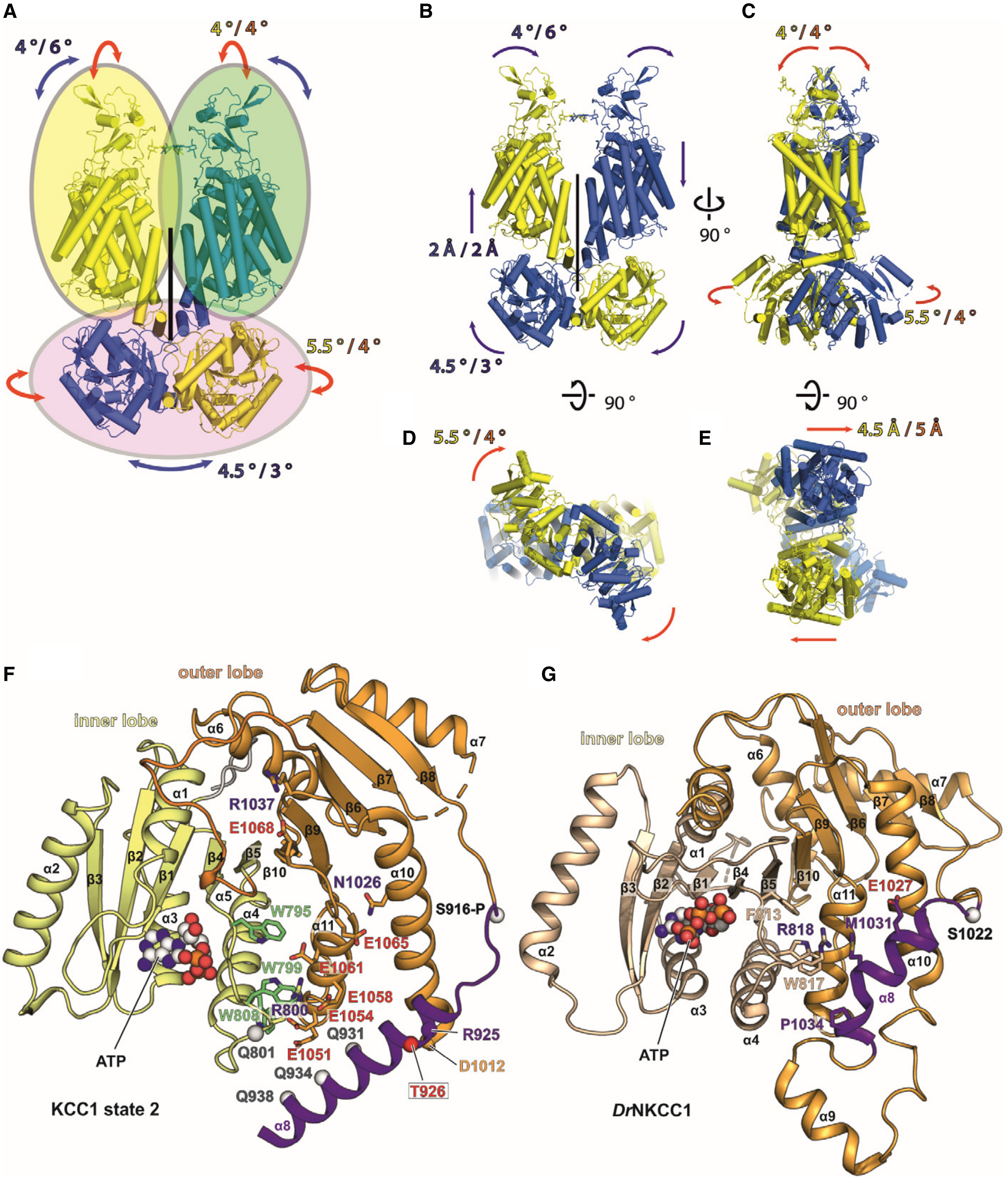

Figure EV3.

 © 2021 The Authors

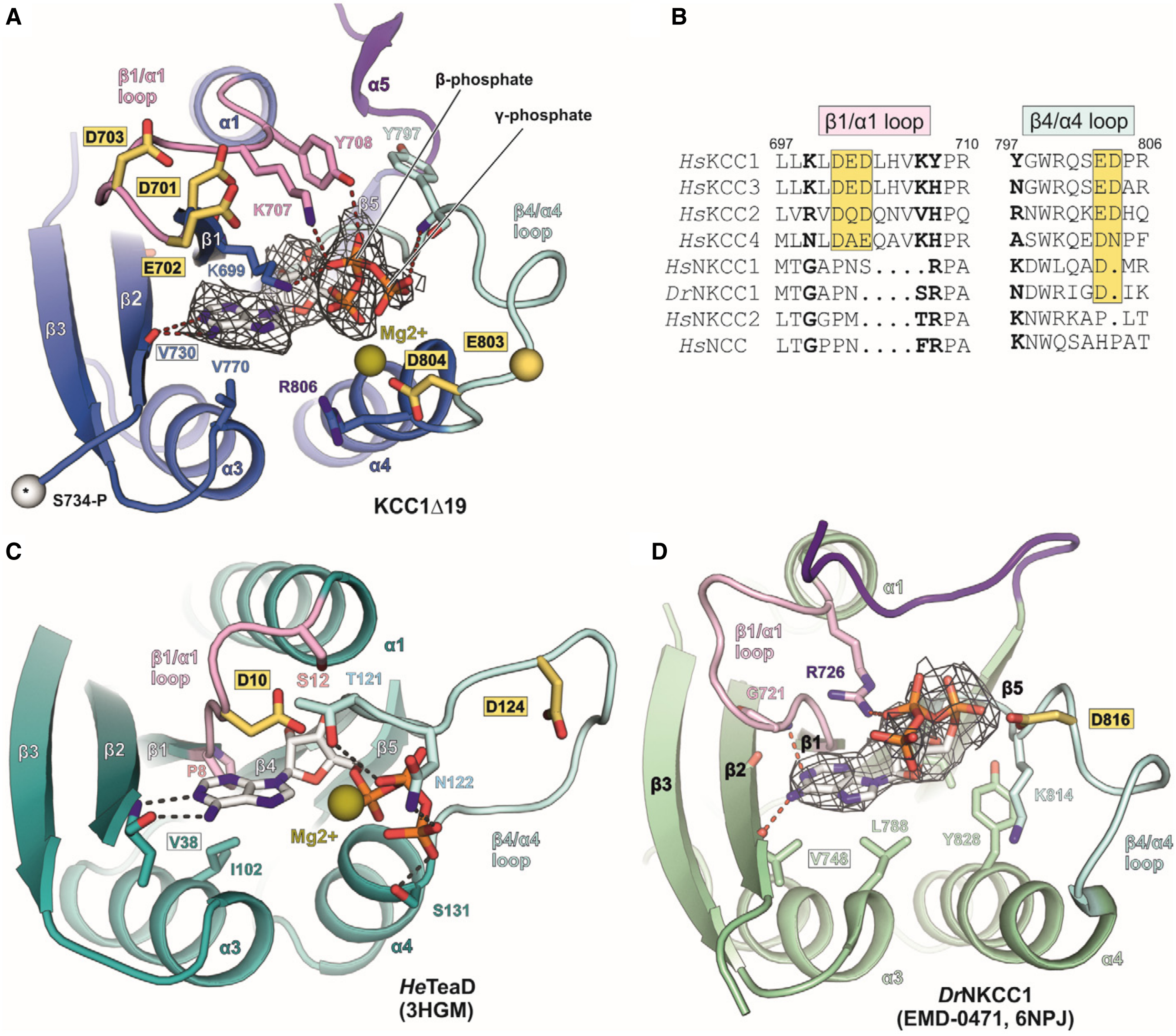

Figure EV4. Comparison of ATP-binding modes in human KCC1Δ19, *Dr*NKCC1 and the ectoine transporter regulatory subunit TeaD from *H. elongata* (Related to Fig 5).

A ATP (stick representation) fitted into EM density map (grey mesh) of human KCC1Δ19. White sphere with asterisk represents the cα atom of phosphorylation site S734-P in KCC1.

B Sequence alignment of β1/α1 and β4/α4 loops for different members of the SLC12 family. Bold letters indicate residues involved in ATP binding. Yellow boxes highlight conserved, acidic residues.

C ATP binding to the regulatory subunit TeaD (pdb: 3HGM) of the ectoine transporter TeaABC from *Halomonas elongata*.

D ATP fitted into unmodelled densities (grey mesh) in the cryo-EM map of *Dr*NKCC1. Labels for conserved valine residues engaged in the main polar backbone interaction with the nucleotide base are framed in a black box. Residues labelled in yellow-highlighted boxes in panels (A–D) are conserved acidic residues in the β1/α1 and β4/α4 loops of the CTD, which could potentially play a role in catalysing ATP hydrolysis, or in Mg^{2+} coordination. Mg^{2+} ions with a potential role for coordinating oxygen atoms in the β- and γ-phosphates of ATP are shown as olive spheres (positions are taken from 3HGM coordinates).

Figure EV5. MD simulation for ATP bound to KCC1 and KCC3 (Related to Fig 6).

A Protein RMSD (teal trace) and ligand RMSD (purple trace) over the course of a 300 ns simulation of ATP bound to the KCC1 dimer (construct Δ19, complete model with 175,573 atoms).

B, C Protein and ligand RMSD over the course of a 500 ns simulation of ATP bound to the isolated CTD of KCC1 (construct Δ19) and KCC3b (construct PM) with 42,461 and 39,805 atoms, respectively.

D, E Ligand-protein contacts for ATP, inferred from a 500 ns MD run of the isolated CTD KCC1 (construct Δ19) (D) and KCC3b (construct PM) (E), respectively. Charged interactions are shown in red (negative) or blue (positive), hydrophobic interactions are shown in green. Further details regarding the nature of protein-ligand interactions are provided in the legend shown in Appendix Fig S12.

© 2021 The Authors

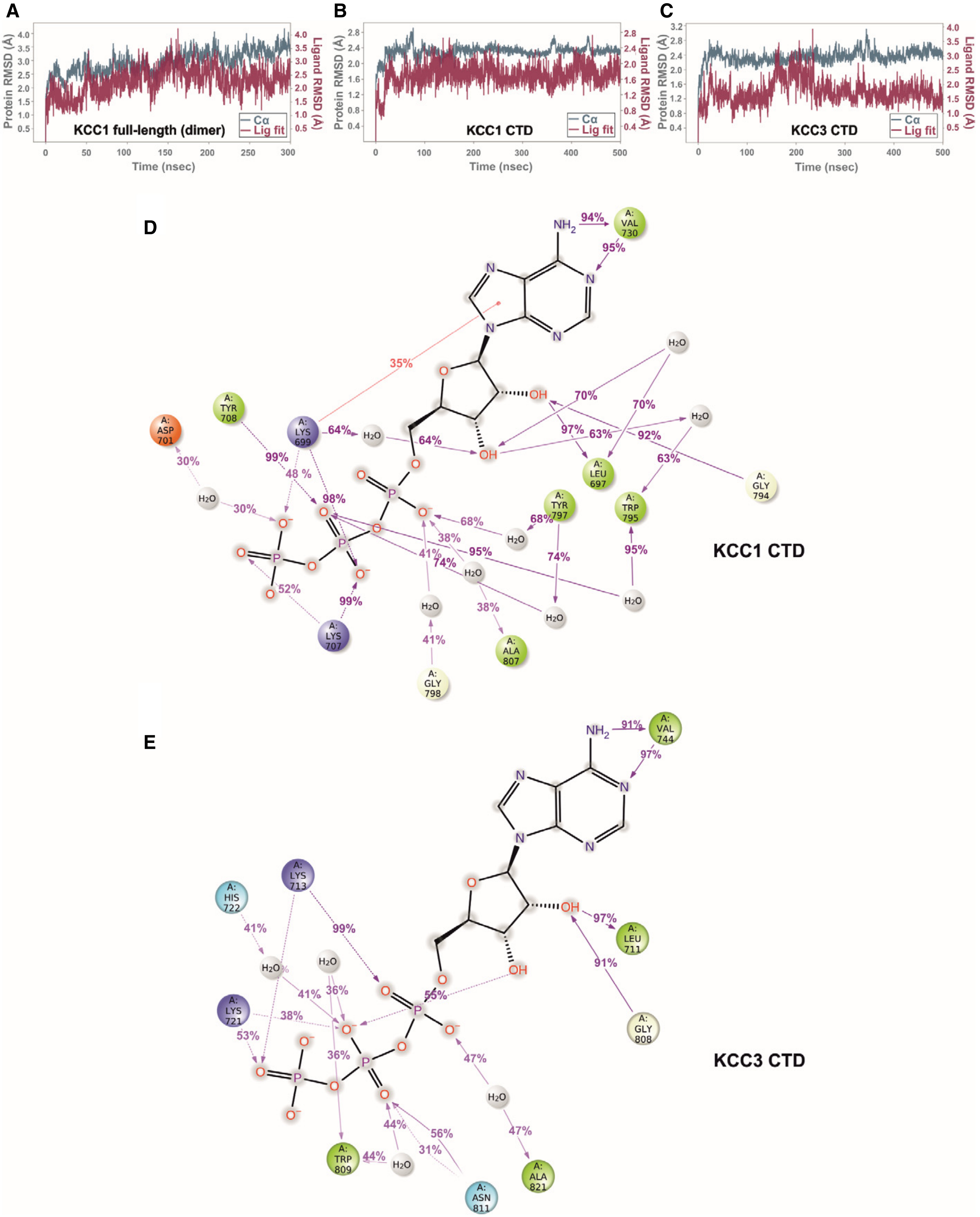

A
B
C
Protein RMSD (Å)
Ligand RMSD (Å)
Time (nsec)
KCC1 full-length (dimer)
KCC1 CTD
KCC3 CTD
Cα
Lig fit
D
KCC1 CTD
E
KCC3 CTD

Figure EV5.
