# Supplementary material for: Phospho‐regulation, nucleotide binding and ion access control in potassium‐chloride cotransporters
Source: EMBO J. 2021 May 25;40(14):e107294. doi: 10.15252/embj.2020107294 (PMC8280820; doi:10.15252/embj.2020107294)
Supplement: Supplementary file 4 — Movie EV2 [file EMBJ-40-e107294-s003.zip › Movie Legend for Movie EV2.docx]

**Extended View Movie Legend for Movie EV2** (related to Figure 3)

Illustration of rigid body movements (mode 1) for KCC1 from 3D variability analysis.
