# Supplementary material for: Phospho‐regulation, nucleotide binding and ion access control in potassium‐chloride cotransporters
Source: EMBO J. 2021 May 25;40(14):e107294. doi: 10.15252/embj.2020107294 (PMC8280820; doi:10.15252/embj.2020107294)
Supplement: Supplementary file 6 — Movie EV4 [file EMBJ-40-e107294-s008.zip › Movie EV4/Movie Legend for Movie EV4.docx]

**Extended View Movie Legend for Movie EV4** (related to Figure 3)

Illustration of rigid body movements (mode 1) for KCC3 from 3D variability analysis.
