# Supplementary material for: Phospho‐regulation, nucleotide binding and ion access control in potassium‐chloride cotransporters
Source: EMBO J. 2021 May 25;40(14):e107294. doi: 10.15252/embj.2020107294 (PMC8280820; doi:10.15252/embj.2020107294)
Supplement: Supplementary file 7 — Movie EV5 [file EMBJ-40-e107294-s009.zip › Movie EV5/Movie Legend for Movie EV5.docx]

**Extended View Movie Legend for Movie EV5** (related to Figure 6).

ATP binding trajectory for KCC3b-PM (CTD only) over a 500 ns MD simulation (run 1, overview of CTD).
