# Supplementary material for: Phospho‐regulation, nucleotide binding and ion access control in potassium‐chloride cotransporters
Source: EMBO J. 2021 May 25;40(14):e107294. doi: 10.15252/embj.2020107294 (PMC8280820; doi:10.15252/embj.2020107294)
Supplement: Supplementary file 8 — Movie EV6 [file EMBJ-40-e107294-s005.zip › Movie EV6/Movie Legend for Movie EV6.docx]

**Extended View Movie Legend for Movie EV6** (related to Figure 6).

Zoomed view of the ATP binding pocket of KCC3b-PM (CTD-only) during a 500 ns MD simulation (run 1).
